# EZH2-H3K27me3-mediated silencing of mir-139-5p inhibits cellular senescence in hepatocellular carcinoma by activating TOP2A

**DOI:** 10.1186/s13046-023-02855-2

**Published:** 2023-11-27

**Authors:** Ke Wang, Xunliang Jiang, Yu Jiang, Jun Liu, Yongtao Du, Zecheng Zhang, Yunlong Li, Xinhui Zhao, Jipeng Li, Rui Zhang

**Affiliations:** 1 Department of digestive surgery, Xijing Hospital, Fourth Military Medical University, Xi’an, 710032 China; 2 Department of General Surgery, Central Theater Command General Hospital of the Chinese People’s Liberation Army, Wuhan, 430064 China; 3 State Key Laboratory of Cancer Biology, Department of Biochemistry and Molecular Biology, Fourth Military Medical University, Xi’an, 710032 China; 4 Department of Hepatobiliary Surgery, XI’AN DAXING hospital, Xi’an, 710032 China; 5 Department of Thyroid and Breast Surgery, Xi’an No.3 Hospital, the Affiliated Hospital of Northwest University, Xi’an, 710018 China; 6 Department of Experimental Surgery, Xijing Hospital, Fourth Military Medical University, Xi’an, 710032 China; 7 State Key Laboratory of Cancer Biology, Department of Immunology, Fourth Military Medical University, Xi’an, 710032 China

**Keywords:** Hepatocellular carcinoma, EZH2, H3K27me3, TOP2A, Cellular senescence

## Abstract

**Background:**

Epigenetic alterations play an important role in hepatocellular carcinoma (HCC) development. Enhancer of zeste homolog 2 (EZH2) is a well-known epigenetic modifier that functions as an oncogene in tumors by promoting the H3K27me3-mediated transcriptional repression of tumor suppressor genes. “Senescent cells” has been proposed as a possible core component of the hallmarks of cancer conceptualization. Induction of cell senescence and targeted elimination of these senescent tumor cells are new strategies for tumor therapy. However, the role of EZH2 in regulating cellular senescence remains poorly understood.

**Methods:**

Bioinformatics analyses suggested that EZH2 and DNA topoisomerase II alpha (TOP2A) are coexpressed in tumors, including HCC. Kyoto Encyclopedia of Genes and Genome (KEGG) pathway enrichment analyses and gene set enrichment analyses (GSEA) suggests a correlation of EZH2 and TOP2A expression with cellular senescence in HCC. MicroRNA (miRNA) inhibitor and mimics, siRNA, PLKO-shRNA, and plenti6.3-miR-139 were used to upregulate or downregulate the expression of target genes. CCK8, EdU, clone formation, and senescence-associated β-galactosidase (SA-β-gal) staining assays were performed to assess cell proliferation and cellular senescence phenotypes. Dual-luciferase reporter and chromatin immunoprecipitation assays were performed to investigate the targeted binding and inhibition of TOP2A 3′ untranslated region (UTR) by miR-139-5p and the DNA enrichment of miR139-5p by EZH2 and H3K27me3. BALB/c nude mice were used to establish a xenograft tumor model and verify the phenotypes upon EZH2 and TOP2A silencing and miR-139 overexpression in vivo. In addition, tissue microarrays were used to analyze the expression patterns and correlations among EZH2, TOP2A, and miR-139-5p expression in HCC.

**Results:**

Bioinformatics analysis revealed that EZH2 and TOP2A are coexpressed in HCC. In vitro gain- and loss-of-function experiments showed that inhibition of EZH2 and TOP2A induces cellular senescence and inhibits proliferation of HCC cells. In vivo tumorigenesis assays indicated that EZH2 and TOP2A knockdown inhibits tumorigenesis by inducing cellular senescence. Mechanistically, EZH2 promotes TOP2A expression by regulating the H3K27me3-mediated epigenetic silencing of miR-139-5p. TOP2A is a direct target of miR-139-5p, and inhibition of miR-139-5p can reverse the promotion by EZH2 of TOP2A expression. The overexpression of miR-139-5p induces cellular senescence and inhibits proliferation of HCC cells both in vitro and in vivo. Clinically, expression of EZH2 and TOP2A are higher in HCC tissues than in normal tissues, and this high coexpression indicates a worse outcome of patients with HCC. Moreover, expression of EZH2 and TOP2A is significantly correlated with tumor differentiation grade, tumor invasion, and TNM stage in HCC. miR-139-5p expression is lower in HCC tumors than in normal tissues and is correlated with better prognosis of HCC patients.

**Conclusions:**

Our study revealed the role of the EZH2/miR-139-5p/TOP2A axis in regulating cellular senescence and cell proliferation in HCC, enriching the molecular mechanisms of EZH2-mediated epigenetic regulation in HCC. Therefore, our results provide insight into the therapeutic potential of targeting EZH2 to induce cellular senescence and then destroy senescent cells for HCC.

**Graphic Abstract:**

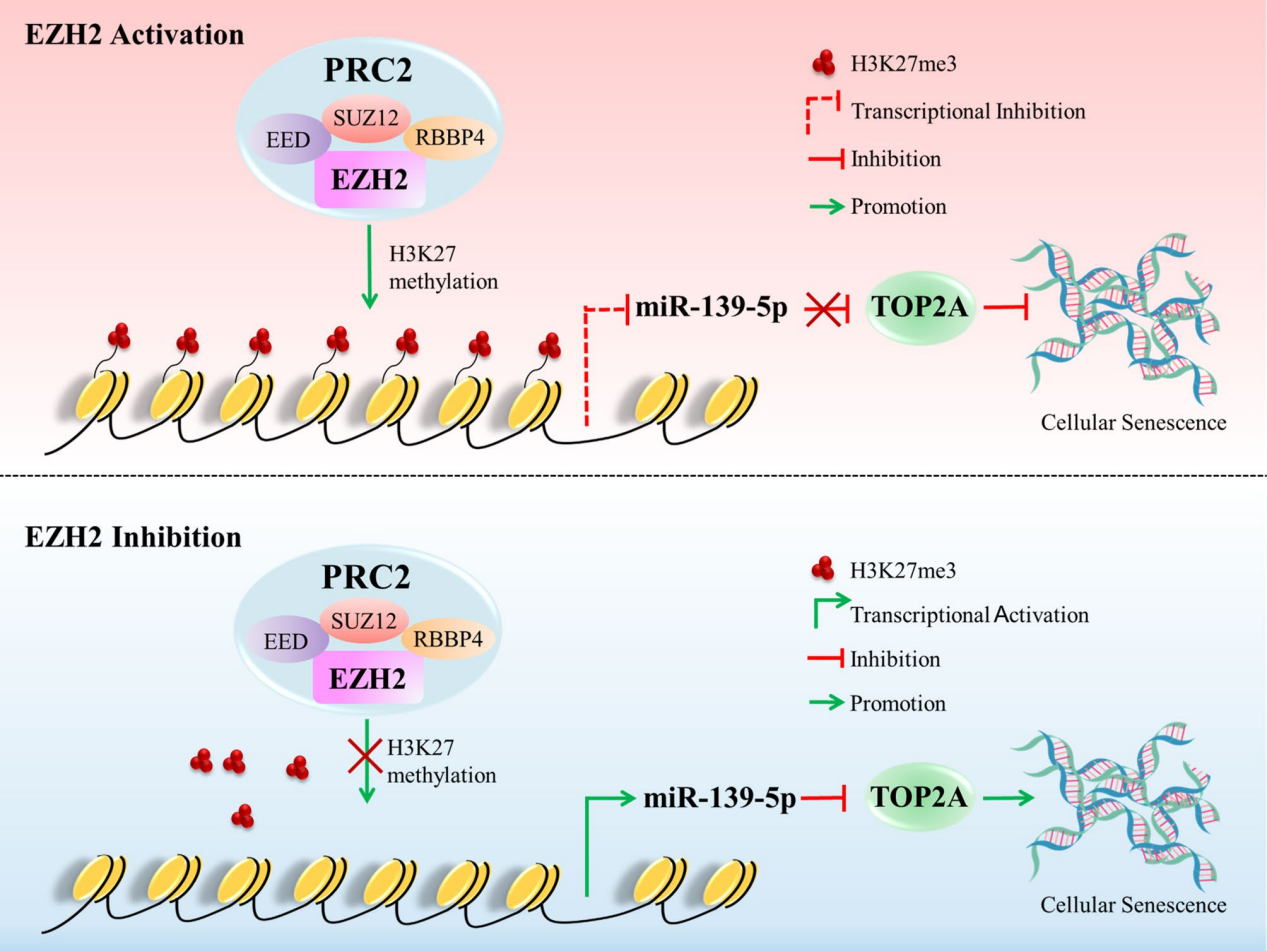

**Supplementary Information:**

The online version contains supplementary material available at 10.1186/s13046-023-02855-2.

## Introduction

Liver cancer is one of the most common malignant tumors worldwide. The incidence of liver cancer continues to increase despite the decline and stabilization of the overall tumor incidence rate [1]. The 5-year relative survival rate for liver cancer from 2010 to 2016 was significantly lower than that for all cancers [1]. The prognosis for HCC, the most common form of primary liver cancer, is poor, and only 5–15% of patients with HCC are eligible for surgical removal. Furthermore, drug resistance and adverse effects limit the effectiveness of chemotherapy for advanced-stage HCC [2]. Epigenetic alterations are common in HCC [3]. Previous studies have reported that epigenetic regulation plays an important role in HCC progression, including the regulation of cell metabolism [4], drug resistance [5], and the tumor microenvironment [6]. Accumulating evidence suggests that targeting epigenetic modifications may be a promising therapeutic strategy for cancer treatment. Thus, investigating the role and molecular mechanisms of epigenetic regulation in HCC will expand our current understanding of its pathogenesis and advance the development of novel therapeutic approaches.

Enhancer of zeste homolog 2 (EZH2) is the enzymatic catalytic subunit of PRC2 and functions as an oncogene in tumors by promoting the H3K27me3-mediated transcriptional repression of tumor suppressor genes [7, 8]. EZH2-mediated epigenetic silencing of miRNAs also contributes to tumor progression [9]. Several EZH2 inhibitors have been reported to display potent antitumor effects [7]. Clinical trials have indicated that targeting EZH2 to treat specific types of cancers, either as monotherapy or as part of combination therapy, is highly promising [10–12]. Epigenetic circuitry containing EZH2 can promote hepatocarcinogenesis and is associated with tumor recurrence and poor survival in HCC [13]. EZH2 promotes stemness and suppresses chemosensitivity in HCC by inducing H3K27me3-mediated transcriptional inhibition of the stemness regulator ATOH8 [14]. Furthermore, EZH2-mediated H3K27me3 on the promoters of CD274 (encoding PD-L1) and interferon regulatory factor 1 (IRF1) inhibits PD-L1 expression in HCC [15]. Considering the important role of EZH2 in regulating malignant biological properties and the immune microenvironment, it may be an ideal therapeutic target for HCC. However, their limited effectiveness and acquired drug resistance still pose challenges to the clinical application of EZH2 inhibitors [12]. Therefore, further investigations into the functions and molecular mechanisms of EZH2 are warranted for its future applications in targeted monotherapies and combination therapies.

By analyzing the genes associated with EZH2 in tumors using the Oncomine dataset, we identified TOP2A as the gene most significantly associated with EZH2 in tumors. TOP2A is responsible for resolving DNA topological strains and controlling genome dynamics, which are essential for maintaining mitotic chromosome structure and genome stability [16]. Aberrant expression of TOP2A is associated with tumor progression, chemotherapy response, and prognosis [17]. Bioinformatic analyses have identified TOP2A as a core oncogene in HCC progression [18, 19]. TOP2A is upregulated and correlated with poor prognosis in HCC [20]. Previous studies have identified EZH2 and TOP2A as comarkers of cancer progression [21, 22]. These findings suggested a positive correlation between EZH2 and TOP2A expression. However, the regulatory mechanisms underlying EZH2 and TOP2A remain unclear.

KEGG pathway enrichment analyses and GSEA of the HCC dataset from The Cancer Genome Atlas (TCGA) revealed a positive correlation of EZH2 and TOP2A expression with cellular senescence. Cellular senescence is widespread across the spectrum of human cancers and senescent cells may be functionally important cell types in the tumor microenvironment [23]. It is a typical irreversible form of proliferation arrest marked by an increase in the number of senescent cells. Senescence can be induced in cells under various conditions, including telomere attrition, genotoxic drugs, irradiation, oncogenic stress, replicative and/or mitotic stress, and epigenetic modifiers [24]. The depletion of TOP2A, a mitotic gene, could induce cellular senescence [25]. Considering the molecular functions of EZH2 and TOP2A, they may play a regulatory role in cellular senescence. However, the mechanism underlying the regulatory role of EZH2 and TOP2A in cellular senescence, particularly in the context of HCC, has not been established.

In this study, we found that inhibition of EZH2 and TOP2A induced cellular senescence and inhibited the proliferation of HCC cells both in vitro and in vivo. Mechanistically, EZH2 activates TOP2A through H3K27me3-mediated transcriptional inhibition of miR-139-5p. Clinically, EZH2 and TOP2A are highly expressed, and miR-139-5p is expressed at low levels in HCC tumor tissues compared with adjacent normal tissues, which indicates a poor prognosis in patients with HCC. This study revealed the role of the EZH2/miR-139-5p/TOP2A axis in promoting HCC progression via the regulation of cellular senescence and suggested that targeting EZH2 to induce senescence and then destroy senescent cells is a promising therapeutic strategy for HCC.

## Materials and methods

### Clinical specimens

Ten pairs of fresh frozen primary tumor tissues and corresponding adjacent normal tissues of HCC patients were obtained from Xijing Hospital (Xi’an, China), with the patients’ informed consent and the approval of the Medical Ethics Committee of First Affiliated Hospital of Fourth Military Medical University (approval number: KY20172013-1).

### Materials

EZH2 inhibitors (EPZ005687 and UNC1999) were purchased from Selleck Chemicals. Rabbit monoclonal antibodies against EZH2 (5246 S), H3K27me3 (9733 S), TOP2A (12,286 S) and Dicer (5362 S) for WB (1:1000), IHC (1:50), and ChIP (1:100) were purchased from Cell Signaling Technology (Cell Signaling Technology, Beverly, MA, USA). The mouse monoclonal antibody against β-actin (A5316) used for WB (1:2000) was purchased from Sigma‒Aldrich (St. Louis, MO, USA). Lipofectamine 2000 transfection reagent was obtained from Invitrogen (Carlsbad, CA, USA).

### Cell lines and cell culture conditions

The human HCC cell lines BEL7404, SMMC7721, SK-Hep1, HepG2, PP5, Huh7, and SW739 and the human normal liver cell line Lo2 were obtained from the Type Culture Collection of the Chinese Academy of Sciences. BEL7404, SMMC7721, SK-Hep1, and SNU739 cells were cultured in RPMI Medium 1640 (Gibco BRL, Grand Island, NY, USA) supplemented with 10% fetal bovine serum (Gibco BRL), penicillin (100 mg/ml) and streptomycin (100 mg/ml). HepG2, PP5, Huh7, and Lo2 cells were cultured in Dulbecco’s Modified Eagle Medium (DMEM, Gibco BRL) supplemented with 10% fetal bovine serum (Gibco BRL), penicillin (100 mg/ml) and streptomycin (100 mg/ml). The human embryonic renal epithelial cell line HEK293T was obtained from ATCC (Manassas, VA, USA) and cultured in Dulbecco’s Modified Eagle Medium (DMEM, Gibco BRL) supplemented with 10% fetal bovine serum (Gibco BRL), penicillin (100 mg/ml), and streptomycin (100 mg/ml). The cells were incubated at 37 °C in a 5% CO2 atmosphere.

### Transfection

Transfection was performed using small interfering RNAs, miRNA mimics, and miRNA inhibitors. When the cells reached approximately 70% coverage, transfection was performed using Lipofectamine 2000 according to the manufacturer’s instructions (Invitrogen). The siRNA duplex oligonucleotides that were used are listed in Table S1.

### Lentivirus packaging and Infection

Lentiviral pLKO.1-shRNA plasmids targeting EZH2 and TOP2A, and lentiviral Plenti6.3-miR139 plasmid expressing miR139-5p were constructed. For lentivirus packaging, pLKO.1-control, pLKO.1-shEZH2, pLKO.1-shTOP2A, Plenti6.3-control, and Plenti6.3-miR139 were transfected into HEK-293T cells using a co-transfection system (pMD2G: psPAX2: target plasmids = 1:3:4) with Lipofectamine 2000. The shRNA sequences were designed by Sigma‒Aldrich (St. Louis, MO, USA) and the Sigma‒Aldrich TRC numbers are as follows: EZH2-sh1: TRCN0000018365; EZH2-sh2: TRCN0000039040; TOP2A-sh1: TRCN0000290998; TOP2A-sh2: TRCN0000049279. The culture supernatant was collected at 48 and 72 h after transfection and then filtered through a 450 nm filter. When cancer cells reached approximately 30% coverage in 6-well plates, 1 ml of lentivirus, 1 ml of fresh medium, and 2 µL of polybrene (Sigma, 10 µg/µl) were added for infection. After incubation for 24 h, puromycin (2 µg/µl) was used for three days to select cells that were successfully infected.

### Western blot

Cells were washed three times with cold phosphate buffered saline (PBS) and harvested using RIPA Lysis Buffer (Shaanxi ZHHC Biotechnology) containing protease inhibitors. The Pierce BCA Protein Assay Kit (Thermo Scientific, USA) was used for total protein quantification. Proteins were separated on 10% SDS polyacrylamide gels and transferred to a nitrocellulose membrane (Millipore). After blocking in 5% nonfat milk for 1 h at room temperature, the membranes were incubated overnight at 4 °C with specific primary antibodies. The membranes were then washed three times with 1x TBST and incubated with horseradish peroxidase (HRP)-conjugated anti-rabbit IgG (1:10,000; PB001; Shaanxi ZHHC Biotechnology) or anti-mouse IgG (1:10,000; PB002; Shaanxi ZHHC Biotechnology) diluted in 1x TBST at room temperature for 60 min. After final washing with 1x TBST, the membranes were visualized using ECL chemiluminescent reagents by Tanon 5500 (Tanon Science & Technology; Shanghai, China).

### Quantitative RT‒PCR

Total RNA was extracted from cells using TRIzol Reagent (Invitrogen), and 1 ug of total RNA was reverse transcribed into cDNA using PrimeScript RT Master Mix (TaKaRa, Tokyo, Japan). Relative mRNA expression was detected using the SYBR Premix Ex Taq reagent kit (Takara, Japan). The fold changes of miR139-5p were normalized to U6, and the fold changes of EZH2/TOP2A/Dicer/p15/p16/p21/EED/SUZ12 were normalized to β-actin using the comparative Ct method (fold change = 2 − ΔΔCt). The primers used for qRT‒PCR are listed in Table S2.

### Cell viability assay

The CCK8 assay was performed to detect cell viability. A total of 2,000 cells from each sample were seeded in 96-well plates, and triplicate wells were prepared for each sample. The supernatants were replaced with 100 µL CCK8 solution (10 µL CCK8:90 µL medium) after the cells were attached and spread, and incubated at 37 °C for 1–4 h. The absorbance at 450 nm was measured using a BIO-RAD Microplate Reader, and the average optical density values were used as statistics. The growth curve was plotted after continuous measurements for five days.

### 5-Ethynyl-20-deoxyuridine (EdU) incorporation and Click-iT ™ reaction

The EdU incorporation assay was performed according to the manufacturer’s instructions (YF 488 Click-iT EdU Imaging Kit, US Everbright, China). For EdU incorporation, 10,000 cells from each sample were seeded in 96-well plates and incubated with EdU solution (50 µM) at 37 °C for 2–4 h. For the click reaction, cells were fixed with 4% paraformaldehyde for 15–30 min, permeabilized with 5% (v/v) Triton-X100 in PBS for 20 min, incubated with 100 µL Click-iT reaction buffer at room temperature in the dark for 30 min, and then incubated with 100 µL 1xHoechst 33,342 in PBS at room temperature away from light for 15–30 min. Images were acquired using an inverted fluorescence microscope. The cells were counted using ImageJ software and the proliferation rates (%) were calculated.

### Plate clone formation assay

For the plate colony formation assay, 1,000 cells were seeded in a 6-well plate and cultivated for 2 weeks. After three washes with PBS, the cells were fixed with 4% paraformaldehyde for 15 min and stained with 0.5% (w/v) crystal violet dye (Sigma‒Aldrich) for 15 min. The dye was washed off, and the plate was dried for imaging. Images of cell clones were captured using an Odyssey Scanner (LI-COR, Lincoln, NE, USA) and counted using the ImageJ software.

### SA-β-gal assay

Senescence-associated β-galactosidase (SA-β-gal) staining was performed according to the manufacturer’s instructions (Senescence β-Galactosidase Staining Kit, C0602, Beyotime, China), and positively stained cells were quantified using ImageJ software.

### Xenograft Tumor model

Female nude mice aged 6–8 weeks were selected for the subcutaneous tumor-bearing assay. All animal procedures were carried out according to the criteria outlined in the Guide for the Care and Use of Laboratory Animals prepared by the National Academy of Sciences and published by the National Institutes of Health (Bethesda, MD, USA) and were approved by the Laboratory Animal Ethics Committee of the Fourth Military Medical University (IACUC-20,171,005). BEL7404 cells stably expressing miR139-5p and shRNAs targeting EZH2 and TOP2A were injected subcutaneously into the flank of each mouse (5 × 10^6^ cells/mouse). BEL7404 cells stably expressing Plenti6.3-control and pLKO.1-control were injected subcutaneously into the flank of each mouse (5 × 10^6^ cells/mouse) for comparison. The maximum (L) and minimum (W) lengths of the tumor were measured using a slide caliper, and the tumor volume was calculated according to the formula V = (L × W^2^)/2. Mice were killed after 4–5 weeks and in vivo solid tumors were dissected and weighed.

### Immunohistochemistry (IHC)

Immunohistochemical staining was performed on formalin-fixed paraffin-embedded tissues. Tissue sections were heated at 60 °C for 1 h and dewaxed and rehydrated. Antigen recovery was performed and the sections were blocked with 3% hydrogen peroxide. Nonspecific staining was blocked with 10% goat serum. Appropriate primary and secondary antibodies were selected, and DAB substrate was used for the chromogenic reaction.

### Tissue microarray assay

Human HCC tissue microarrays, containing 47 tumor tissue samples and 47 adjacent normal tissue samples, were obtained from the Department of Pathology, Fourth Military Medical University (Xi’an, China). The slides were stained with anti-human EZH2 (Cell Signaling Technology, 5246 S, 1:50) and TOP2A (Cell Signaling Technology, 12,286 S, 1:50) antibodies. The slides were scanned using a Pannoramic MIDI (Santa Clara, CA, USA) and quantified using the Quant Center. Correlation of protein expression was analyzed using GraphPad Prism (Version 5; La Jolla, CA, USA).

### Chromatin immunoprecipitation (ChIP) assay

ChIP analysis of EZH2/H3K27me3 was performed using the SimpleChIP Enzymatic Chromatin IP Kit (Cell Signaling Technology, 9003) according to the manufacturer’s instructions. The primer sequences used for the ChIP analysis are listed in Table S2.

### Dual luciferase reporter assay

Cells were seeded in 48-well plates and co-transfected with pGL3-TOP2A 3’-UTR, NC mimics/miR139-5p mimics, and pRL-TK using Lipofectamine 2000 (Invitrogen) for 48 h. The supernatant of lysed cells was used to measure luciferase activity using the Dual Luciferase Reporter Assay System (Promega, USA). The relative firefly luciferase activity was normalized to Renilla luciferase activity.

### Public database

The genes correlated with EZH2 in cancers and the expression of EZH2 and TOP2A in HCC cancer tissues and normal tissues were analyzed using the Oncomine database (https://www.oncomine.org/). EZH2 and TOP2A expression profiles were analyzed using Gene Expression Profiling Interactive Analysis (GEPIA, http://gepia.cancer-pku.cn). Enrichment of the TOP2A promoter region by EZH2 and H3K27me3 was analyzed using chromatin immunoprecipitation (ChIP)-sequencing databases (https://www.encodeproject.org/). The differentially expressed genes in HCC with high and low expression of EZH2 and TOP2A and the correlation between EZH2 and TOP2A expression and the clinicopathological features of HCC patients were analyzed using Assistant for Clinical Bioinformatics (https://www.aclbi.com/static/index.html#/). GSE185913 in the GEO database (https://www.ncbi.nlm.nih.gov/geo/), TargetScan Human 7.2 (http://www.targetscan.org/vert_71/), and miRcode database (http://mircode.org/) were used to screen miRNAs that target TOP2A in HCC. Correlation analyses of EZH2/TOP2A and miRNAs and miRNA expression in HCC were performed using StarBase v2.0 (http://starbase.sysu.edu.cn/). The prognostic analyses of HCC were performed using Kaplan–Meier Plotter (http://kmplot.com/analysed/), and the auto-select best cutoff value was chosen. The variable values were iterated over from the lower quartile to the upper quartile, each setting was calculated by Cox regression, and the most significant cutoff value was used as the best cutoff to divide the input data into two groups [26].

### Statistical analysis

Statistical analyses were performed using GraphPad Prism, version 8.0. Significance between two groups was analyzed using a two-tailed unpaired or paired Student’s t test. The significance of more than three groups with one variable was analyzed using a one-way ANOVA. The significance of groups with two variables was analyzed using a two-way ANOVA. The significance of the growth curves was analyzed using a two-way ANOVA. The treatment groups were compared to the control group unless stated otherwise. The correlation between the mRNA and protein levels of EZH2, TOP2A, and miR139-5p was analyzed using linear regression analysis. Prognostic analysis was performed using the Kaplan‒Meier method and log-rank test. n = 3 independent experiments unless stated otherwise. The data are presented as the means ± SD, unless indicated otherwise. Differences were considered statistically significant when p < 0.05 (*), p < 0.01 (**) or p < 0.001 (***).

## Results

### Coexpression of EZH2 and TOP2A in HCC

**Fig. 1:**
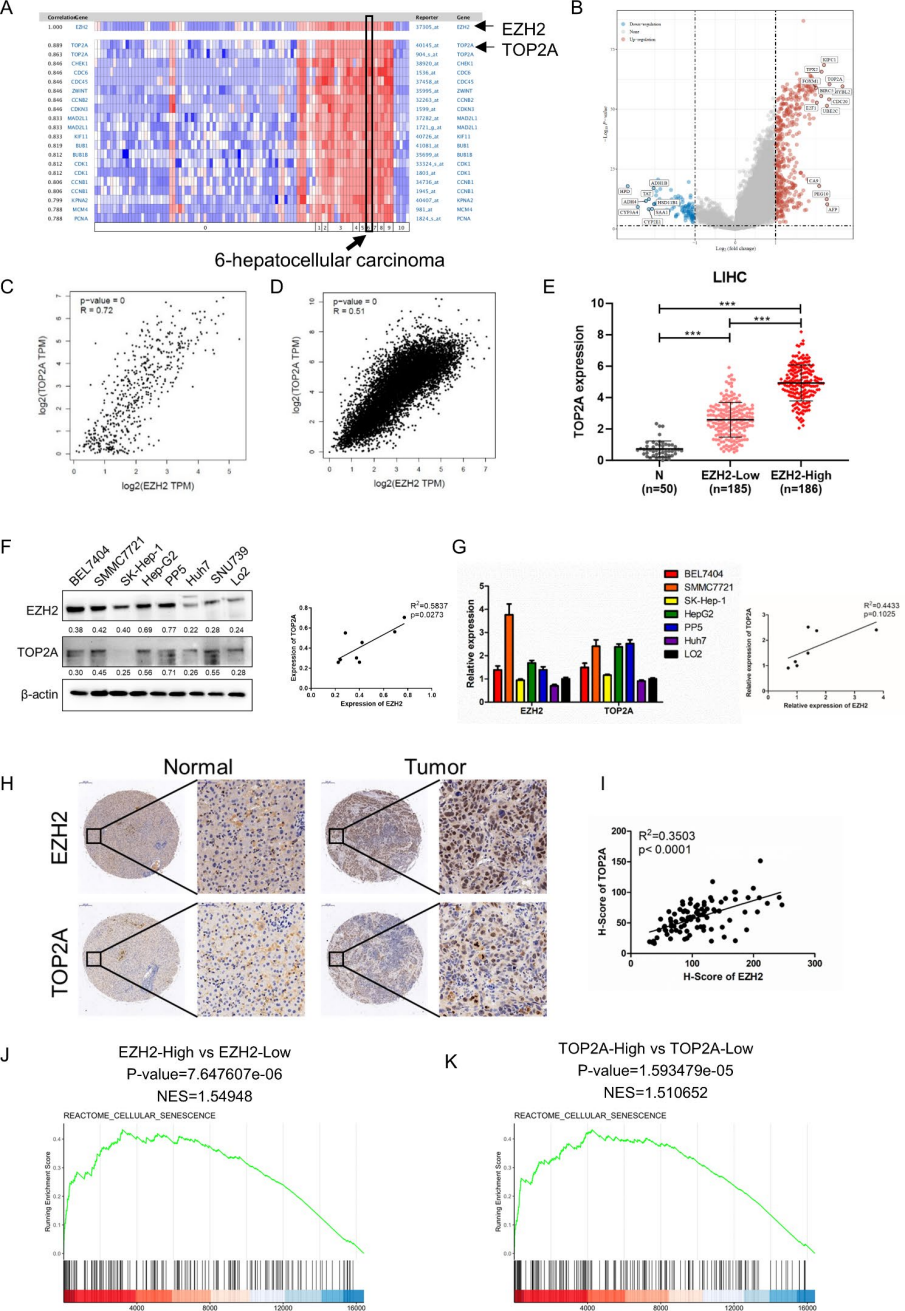
Coordinated expression of EZH2 and TOP2A in HCC. **(A)** Heatmap of genes correlated with EZH2 in cancers analyzed by the Oncomine database. **(B)** Volcano plot showing the differentially expressed genes of EZH2 in HCC by reanalyzing the RNA-seq data in the TCGA dataset using the limma package in R software. (**C** and **D**) Correlation analysis of EZH2 and TOP2A in HCC **(C)** and 31 tumors (including ACC, BLCA, BRCA, CESC, CHOL, COAD, DLBC, ESCA, GBM, HNSC, KICH, KIRC, KIRP, LAML, LGG, LIHC, LUAD, LUSC, MESO, OV, PAAD, PCPG, PRAD, READ, SARC, SKCM, STAD, TGCT, THCA, THYM, UCEC, UCS, and UVM) in TCGA **(D)**. **(E)** TOP2A expression in normal tissues (n = 50), HCC tissues with low EZH2 expression (n = 185), and HCC tissues with high EZH2 expression (n = 186) by reanalyzing the RNA-seq data of HCC in the TCGA dataset using R software v4.0.3. **(F)** Western blot and **(G)** RT-qPCR analysis of EZH2 and TOP2A expression patterns in HCC cell lines. **(H)** Protein levels of EZH2 and TOP2A in HCC tissues and paired paracancerous tissues (n = 47) analyzed by IHC. GSEA of RNA-seq data from TCGA of EZH2 high expression versus EZH2 low expression **(J)** and TOP2A high expression versus TOP2A low expression using the Reactome cellular senescence gene set annotated in R-HSA-2,559,583. NES, normalized enrichment score. *p < 0.05, **p < 0.01, ***p < 0.001

To identify genes correlated with EZH2 in HCC, we performed a correlation gene analysis using data from the Oncomine database. The results showed that TOP2A was the gene most closely associated with EZH2 in various cancer types, including HCC (Fig. 1A). We then analyzed the differentially expressed genes in HCC with high and low EZH2 expression. The volcano plot showed that TOP2A was upregulated in high-EZH2 HCC (Fig. 1B). We further analyzed the correlation between EZH2 and TOP2A expression in cancer. EZH2 was positively correlated with TOP2A in HCC (Fig. 1C) and 31 tumors in the TCGA database (Fig. 1D). We reanalyzed the RNA-seq data of the HCC dataset in TCGA and found that TOP2A was more highly expressed in high-EZH2 HCC tissues than in low-EZH2 HCC and normal tissues (Fig. 1E). We assessed the protein and mRNA levels of EZH2 and TOP2A in various HCC cell lines and found a positive correlation between EZH2 and TOP2A (Fig. 1F and G). Moreover, we examined the protein expression levels of EZH2 and TOP2A in HCC tissues by immunohistochemistry (IHC) using a tissue microarray and verified the positive correlation between EZH2 and TOP2A in HCC (Fig. 1H and I). To further confirm the underlying functions of EZH2 and TOP2A, we analyzed the differentially expressed genes of TOP2A in HCC (Fig. S1A) and performed functional enrichment of the RNA-seq data of EZH2 and TOP2A using KEGG enrichment analysis. These results indicated that the functional enrichment of the two genes was particularly consistent, and cellular senescence aroused our research interest (Fig. S1B). In support of this notion, we performed GSEA on the TCGA HCC dataset and found an enrichment of senescence signatures in groups with high EZH2 and TOP2A expression (Fig. 1J K, and S1C). Collectively, these findings indicate that EZH2 and TOP2A are coexpressed in HCC and may be involved in the regulation of cellular senescence.

### EZH2 promotes proliferation and inhibits senescence of HCC cells

**Fig. 2:**
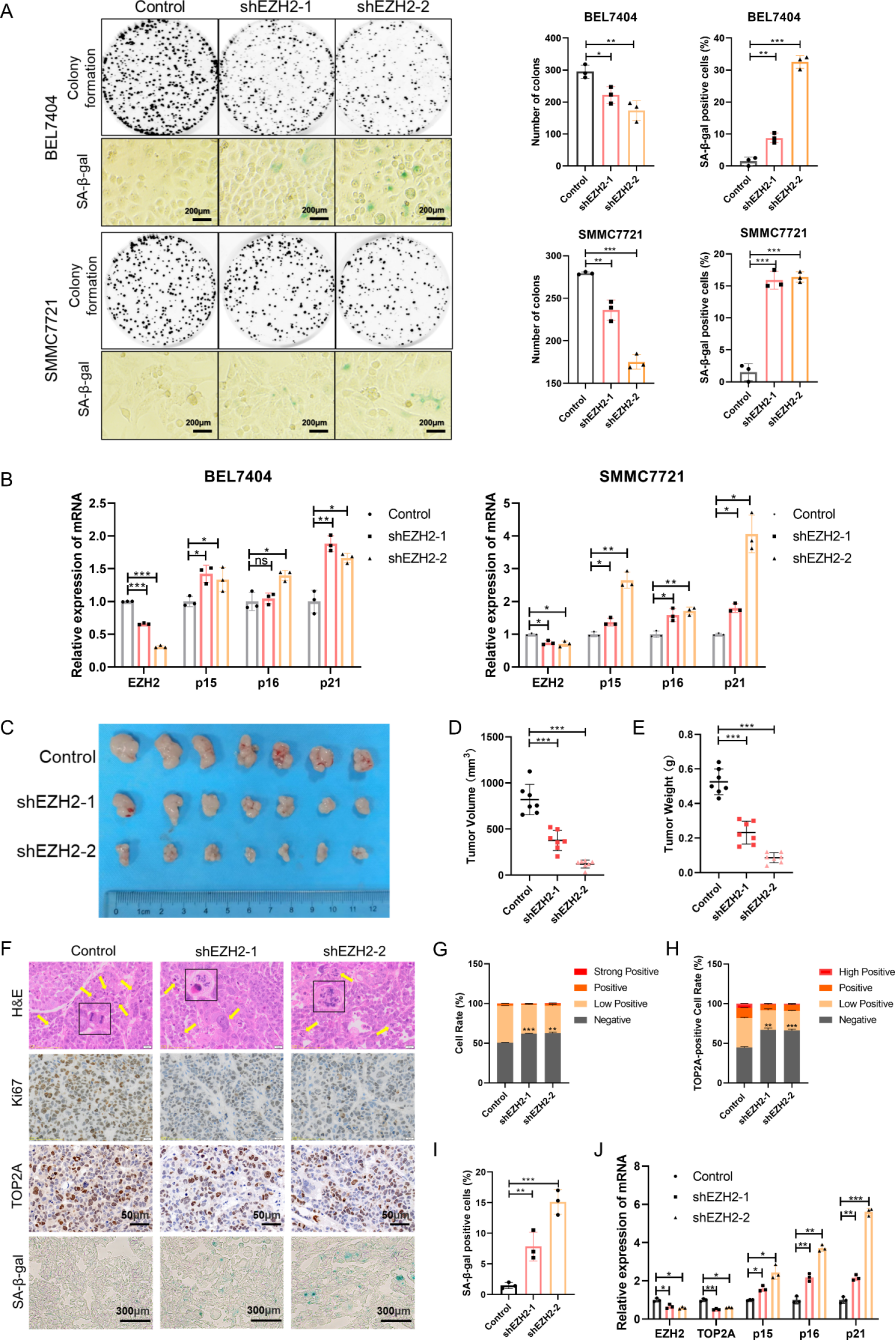
Inhibition of EZH2 impairs growth and induces senescence of HCC cells both in vitro and in vivo. **(A)** Representative images of colony formation and SA-β-gal staining of cells. **(B)** mRNA levels of EZH2 and senescence markers p15, p16, and p21 in BEL7404 and SMMC7721 cells. **(C)** Representative images of tumors in nude mice subcutaneously inoculated with BEL7404 cells stably expressing pLKO.1-shEZH2s and pLKO.1-control (seven mice in each group). (**D** and **E**) The tumor volume **(D)** and weight **(E)** in nude mice subcutaneously inoculated with BEL7404 cells stably expressing pLKO.1-shEZH2s and pLKO.1-control. **(F)** Representative images of H&E, Ki67, TOP2A, and SA-β-gal staining of tumor tissues. **(G)** The cell rate of Ki67-positive cells in tumors. **(H)** The cell rate of TOP2A-positive cells in tumors. **(I)** The cell rate of SA-β-gal-positive cells in tumors. **(J)** mRNA levels of EZH2, TOP2A, and senescence markers p15, p16, and p21 in tumors in nude mice subcutaneously inoculated with BEL7404 cells. *p < 0.05, **p < 0.01, ***p < 0.001

Although the carcinogenic function of EZH2 in HCC is well established, its effect on cellular senescence and the underlying mechanism remains unclear. To investigate the effect of EZH2 on HCC cell growth and senescence, we used EZH2 inhibitors to interfere with its enzyme activity and siRNAs and shRNAs to decrease its expression in the BEL7404 and SMMC7721 cell lines. Western blotting verified that EZH2 was knocked down using targeted siRNAs and shRNAs (Fig. S2A and S2B). The results of the CCK8 and EdU assays showed that the proliferative activity of the two HCC cell lines decreased with EZH2 inhibitor treatment (Fig. S2C and S2D). Similarly, cell proliferation slowed down after EZH2 knockdown by siRNAs (Fig. S2E and S2F). We then performed plate colony formation and senescence-associated β-galactosidase (SA-β-gal) staining assays using the BEL7404 and SMMC7721 cell lines with stable knockdown of EZH2. The results showed that colony formation was inhibited and the SA-β-gal–positive cell rate increased with EZH2 knockdown (Fig. 2A). Morphologically, the cells exhibited a senescent phenotype; that is, the cells became flatter and enlarged after EZH2 knockdown (Fig. 2A). Moreover, we tested the mRNA levels of the senescence molecular markers p15, p16, and p21 and found that the knockdown of EZH2 upregulated them (Fig. 2B). To further verify these results in vivo, we constructed a xenograft tumor model in nude mice using BEL7404 cells with stable knockdown of EZH2. EZH2 knockdown suppressed tumor growth (Fig. 2C). Compared to the control group, the tumor volume and weight decreased in the EZH2 knockdown groups (Fig. 2D and E). We then performed hematoxylin and eosin (H&E), Ki67, TOP2A and SA-β-gal staining of tumor tissues (Fig. 2F). EZH2 loss resulted in increased abnormal cell division, decreased Ki67 and TOP2A expression, and increased SA-β-gal expression in tumors (Fig. 2G-I). Moreover, the molecular markers of senescence were upregulated in EZH2 knockdown tumors (Fig. 2J). These data suggest that EZH2 promotes cell growth and inhibits cellular senescence in HCC cells.

### TOP2A promotes proliferation and inhibits senescence in HCC cells

**Fig. 3:**
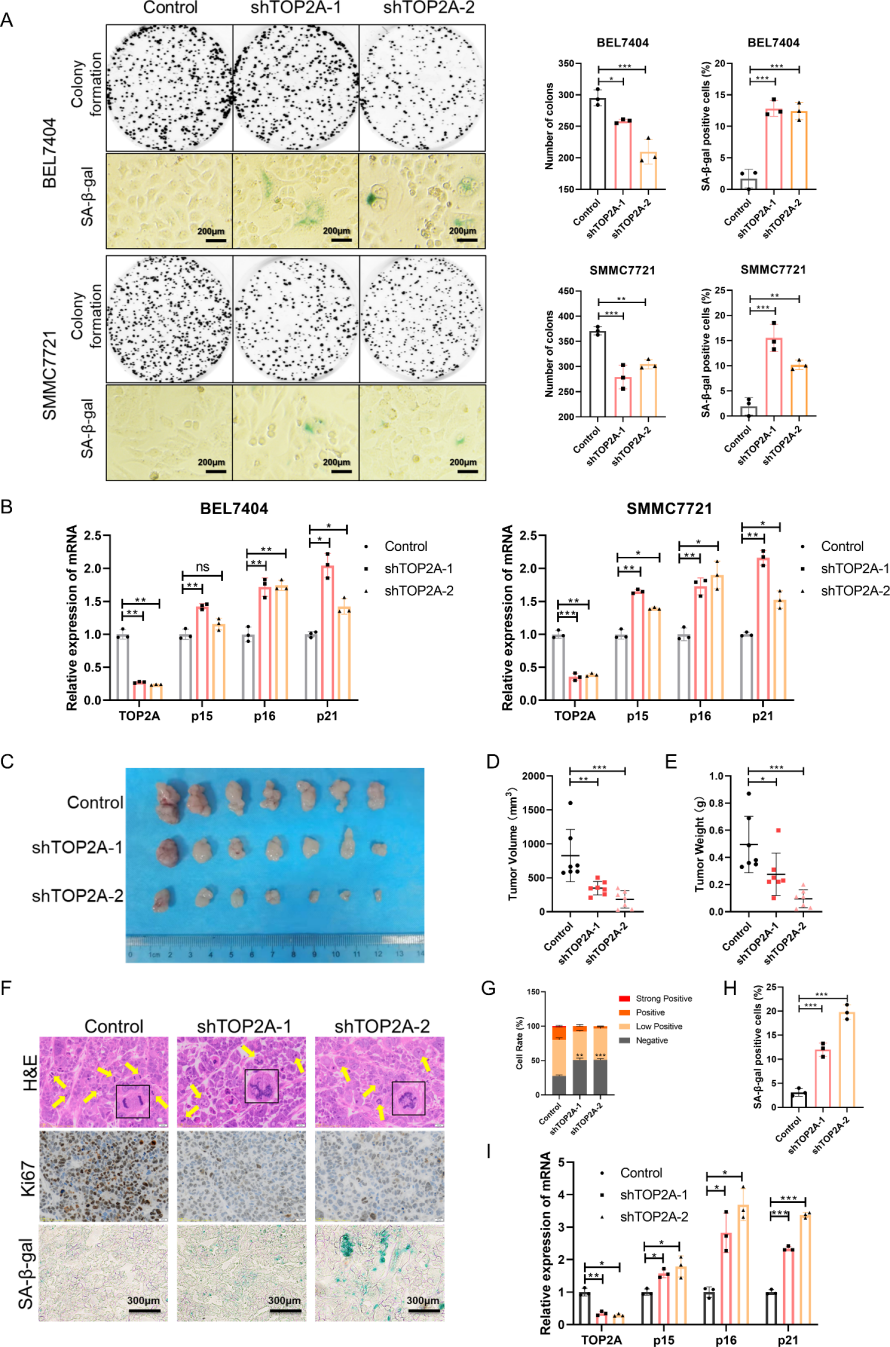
TOP2A knockdown impairs cell growth and induces cellular senescence in HCC. **(A)** Representative images of colony formation and SA-β-gal staining of cells stably expressing pLKO.1-control and pLKO.1-shTOP2As. **(B)** mRNA levels of TOP2A and senescence markers p15, p16, and p21 in BEL7404 and SMMC7721 cells. **(C)** Representative images of tumors in nude mice subcutaneously inoculated with BEL7404 cells stably expressing pLKO.1-shTOP2As and pLKO.1-control (seven mice in each group). (**D** and **E**) The tumor volume **(D)** and weight **(E)** in nude mice subcutaneously inoculated with BEL7404 cells stably expressing pLKO.1-shTOP2As and pLKO.1-control. **(F)** Representative images of H&E, Ki67, and SA-β-gal staining of tumor tissues. **(G)** The cell rate of Ki67-positive cells in tumors. **(H)** The cell rate of SA-β-gal-positive cells in tumors. **(I)** mRNA levels of TOP2A and senescence markers p15, p16, and p21 in tumors in nude mice subcutaneously inoculated with BEL7404 cells. *p < 0.05, **p < 0.01, ***p < 0.001

TOP2A is a key driver of the compaction of nuclear DNA into individualized X-shaped chromosomes during preparation for mitosis [27]. The dysfunction of TOP2A is related to genome instability, cell cycle arrest, and DNA damage, which trigger cell growth arrest and cellular senescence [28]. To study the role of TOP2A in HCC cell proliferation and senescence, we used targeted siRNAs and shRNAs to downregulate TOP2A. Knockdown of TOP2A using siRNAs inhibited the proliferation of BEL7404 and SMMC7721 cells (Fig. S3A-S3D). Knockdown of TOP2A using shRNAs inhibited colony formation and increased the SA-β-gal–positive cell rate in HCC cells (Fig. S3E and 3 A). Moreover, compared to the control groups, the mRNA levels of p15, p16, and p21 were upregulated in the EZH2 knockdown groups (Fig. 3B). To further confirm these results in vivo, we constructed a xenograft tumor model in nude mice using BEL7404 cells stably expressing TOP2A-targeted shRNAs. In vivo growth of xenografts derived from cancer cells stably expressing TOP2A shRNA was significantly suppressed (Fig. 3C). The tumor volume and weight decreased with TOP2A knockdown (Fig. 3D and E). We then performed H&E, Ki67, and SA-β-gal staining of tumor tissues (Fig. 3F). Compared with control tumors, TOP2A knockdown tumors exhibited increased abnormal cell division, decreased Ki67 expression, and an increased SA-β-gal–positive cell rate (Fig. 3G H). Moreover, TOP2A knockdown resulted in the upregulation of molecular markers of senescence in tumors (Fig. 3I). Taken together, these results indicate that TOP2A knockdown impairs cell growth and induces cellular senescence in HCC.

### TOP2A is a direct target of miR-139-5p

**Fig. 4:**
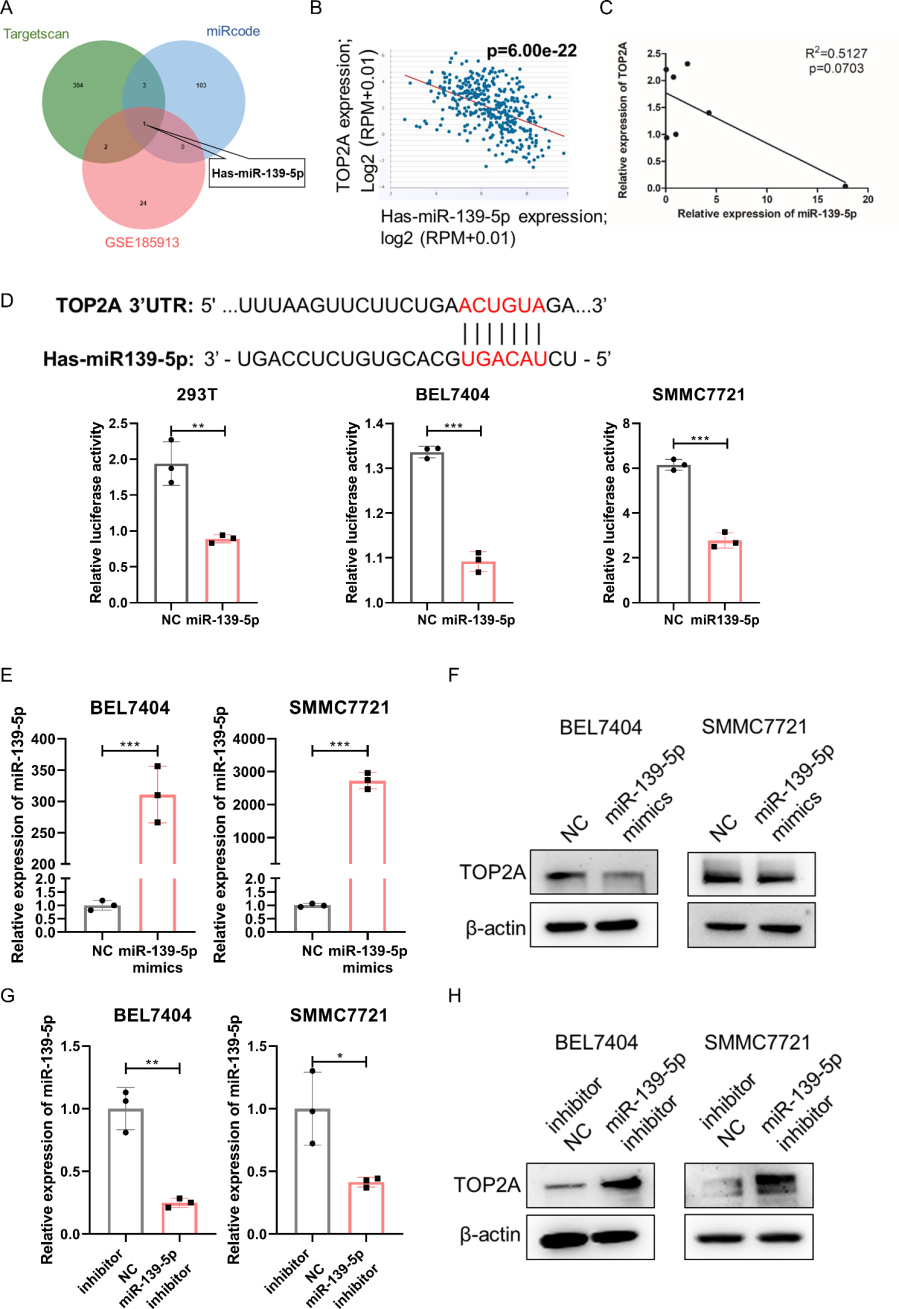
TOP2A is a direct target of miR-139-5p. **(A)** Overlap of miRNAs differentially expressed in hepatocellular carcinoma and adjacent normal tissue in the GSE185913 set and miRNAs targeting TOP2A in the TargetScan and miRcode databases. **(B)** Correlation analysis of miR-139-5p and TOP2A in HCC using starBase v2.0. **(C)** Correlation analysis of miR-139-5p and TOP2A in HCC cell lines. **(D)** Putative binding site of miR-139-5p in the TOP2A 3’UTR and dual luciferase reporter assay of miR-139-5p and TOP2A 3’UTR in HEK293T, BEL7404 and SMMC7721 cell lines. **(E)** RT‒qPCR of miR-139-5p in BEL7404 and SMMC7721 cells transfected with miR-139-5p mimics. **(F)** Western blot of TOP2A in BEL7404 and SMMC7721 cells transfected with miR-139-5p mimics. **(G)** RT‒qPCR of miR-139-5p in BEL7404 and SMMC7721 cells transfected with miR-139-5p inhibitor. **(H)** Western blot of TOP2A in BEL7404 and SMMC7721 cells transfected with miR-139-5p inhibitor. *p < 0.05, **p < 0.01, ***p < 0.001

To investigate the regulatory mode of EZH2 on TOP2A, we analyzed the chromatin immunoprecipitation sequencing (ChIP-seq) data of EZH2 and H3K27me3 in the HepG2 and MCF-7 cell lines from the ENCODE database, with the ChIP-seq data of FOXM1 as a positive control. We found no EZH2 and H3K27me3 binding peaks in the promoter region of TOP2A (Fig. S4). Thus, TOP2A transcription is not directly regulated by EZH2. miRNAs can function as bridges in transcriptional regulation by EZH2 [9]. Therefore, we speculate that EZH2 indirectly regulates TOP2A expression via miRNAs. To identify potential miRNAs that target TOP2A in HCC, we predicted candidate miRNAs targeting TOP2A using the TargetScan and miRcode databases. We then analyzed the data derived from the GSE185913 dataset and found 27 differentially expressed miRNAs in normal and liver cancer tissues. Overlap of the three datasets revealed that miR-139-5p could be a miRNA that targets TOP2A in HCC (Fig. 4A). Correlation analysis of data in the starBase v2.0 database and expression in HCC cell lines revealed a negative correlation between TOP2A and miR-139-5p (Fig. 4B C). To further confirm whether TOP2A is a direct target of miR139-5p, we cloned the 3’-UTR of TOP2A, encompassing the target site downstream of the firefly luciferase gene. The dual luciferase reporter assay showed that compared with the negative control (NC), transfection with miR139-5p mimics significantly inhibited luciferase reporter activity (Fig. 4D), confirming that miR139-5p directly targets the 3′UTR of TOP2A. Moreover, the expression of TOP2A was repressed by the ectopic expression of miR139-5p mimics but was promoted by transfection with miR139-5p inhibitors (Fig. 4E H). Collectively, these results indicate that miR-139-5p directly targets TOP2A and inhibits its expression in HCC cells.

### EZH2 promotes TOP2A expression by epigenetically silencing miR-139-5p

**Fig. 5:**
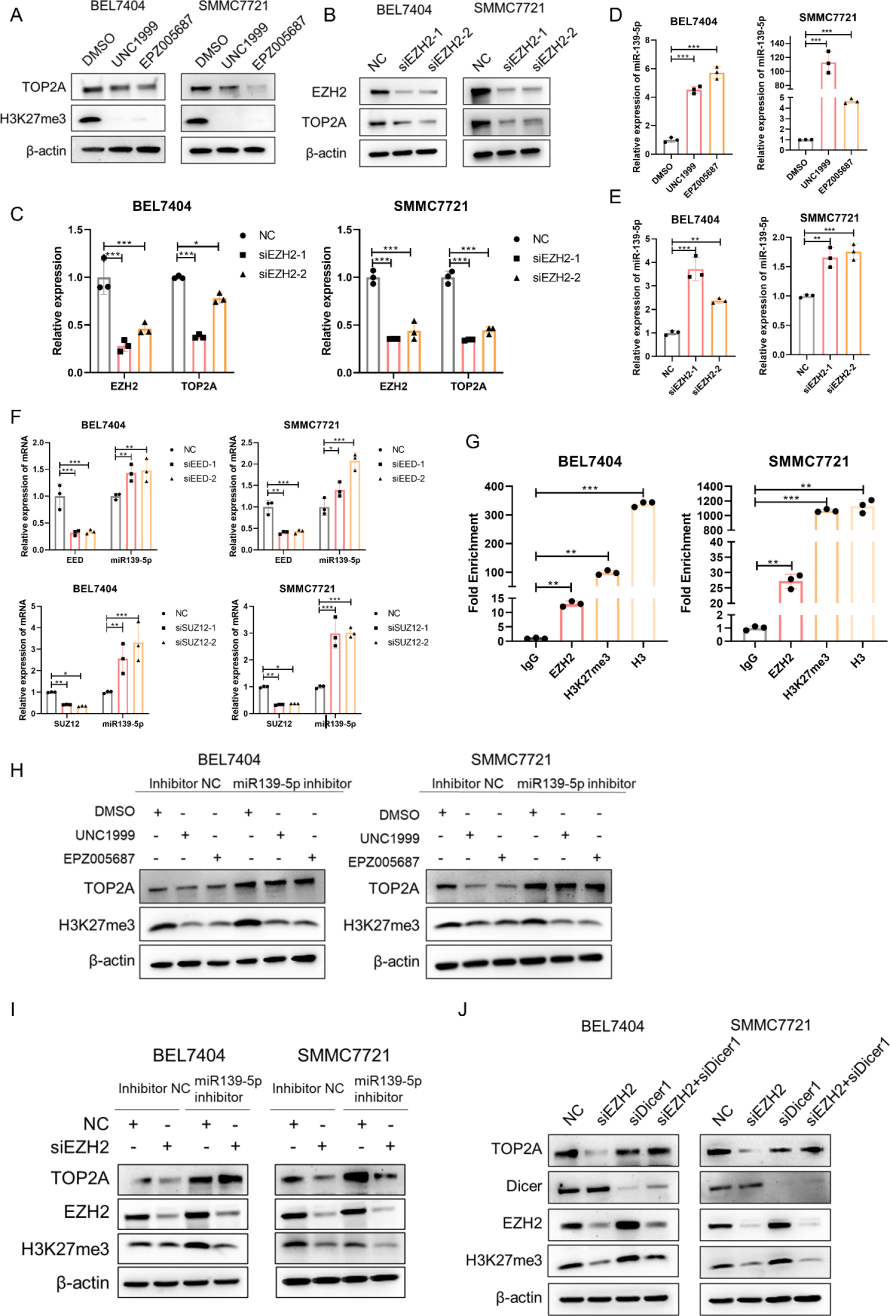
EZH2 acts as a positive regulator of TOP2A by promoting H3K27me3-mediated epigenetic silencing of miR-139-5p. **(A)** Protein levels of TOP2A and H3K27me3 in cells treated with the EZH2 inhibitors UNC1999 and EPZ005687. (**B** and **C**) Protein levels **(B)** and mRNA levels **(C)** of EZH2 and TOP2A in cells transfected with EZH2-targeted siRNAs. (**D** and **E**) mRNA levels of miR-139-5p in cells treated with EZH2 inhibitors UNC1999 and EPZ005687 **(D)** and in cells transfected with EZH2 targeted siRNAs **(E)**. **(F)** mRNA levels of miR-139-5p in cells transfected with EED- and SUZ12- targeted siRNAs. **(G)** ChIP‒qPCR analysis of the enrichment of EZH2 and H3K27me3 on the promoter region of miR-139-5p in HCC cell lines. IgG was used as a negative control and H3 was used as a positive control. **(H)** Western blot analysis of TOP2A and H3K27me3 in HCC cells transfected with inhibitor NC and miR139-5p inhibitor and treated with DMSO, UNC1999, or EPZ005687. **(I)** Western blot analysis of TOP2A, EZH2 and H3K27me3 in HCC cells transfected with inhibitor NC and miR139-5p inhibitor and transfected with NC or siEZH2. **(J)** Western blot analysis of TOP2A, Dicer, EZH2 and H3K27me3 in HCC cells transfected with NC, siEZH2, siDicer, and siEZH2 and siDicer. *p < 0.05, **p < 0.01, ***p < 0.001

As shown in Fig. 5A C and supplemental Fig. 5A, TOP2A expression was repressed by EZH2 inhibitor-mediated H3K27me3 inhibition and EZH2 siRNA-mediated EZH2 downregulation. However, miR-139-5p expression was promoted by EZH2 inhibitor treatment and EZH2 downregulation (Fig. 5D and E). Furthermore, knockdown of the PRC2 complex subunits EED and SUZ12 also resulted in the downregulation of TOP2A and upregulation of miR-139-5p (Fig. S5B and 5 F). To further confirm the epigenetic regulation of EZH2 on miR-139-5p, we performed a chromatin immunoprecipitation (ChIP) assay and found a significant enrichment of EZH2 and H3K27me3 on the promoter region of miR-139-5p in BEL7404 and SMMC7721 cells (Fig. 5G). To investigate whether EZH2 promotes TOP2A expression via miR-139-5p, we performed rescue assays. The decrease in TOP2A protein expression induced by the EZH2 inhibitors UNC1999 and EPZ005687 was abrogated by miR-139-5p inhibition (Fig. 5H). Similarly, the reduction in TOP2A mRNA and protein levels caused by EZH2 knockdown was rescued by miR-139-5p inhibition (Fig. 5I and S5D). Previous studies have reported that Dicer is essential for the maturation of most miRNAs [29]. As shown in Fig. 5J, S5E, and S5F, Dicer depletion blocked the reduction in TOP2A mRNA and protein expression induced by EZH2 knockdown. Taken together, EZH2 promoted TOP2A expression by inducing H3K27me3-mediated epigenetic silencing of miR-139-5p.

### MiR-139-5p inhibits proliferation and induces senescence in HCC cells

**Fig. 6:**
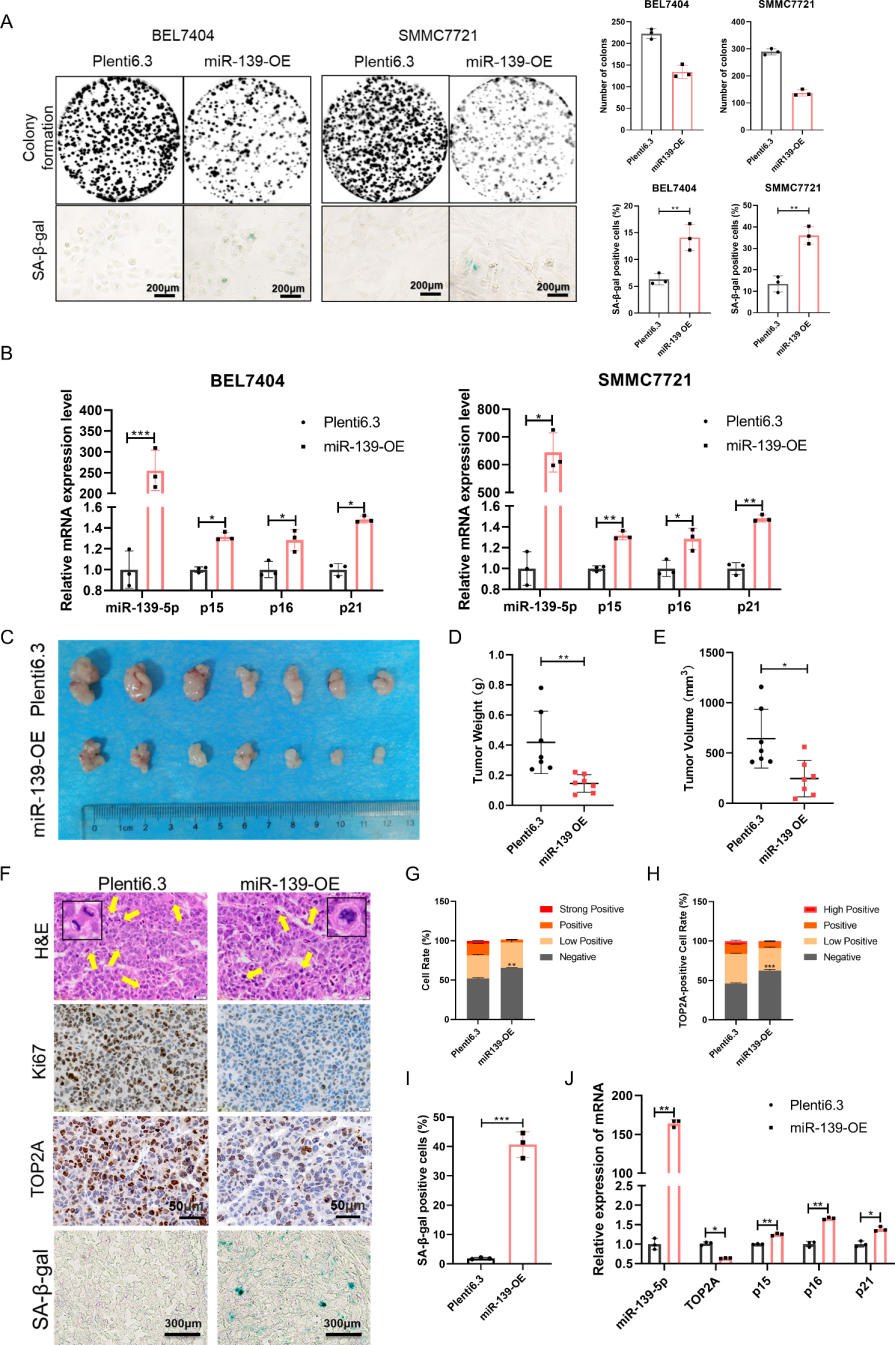
miR-139-5p inhibits cell proliferation and induces senescence in HCC. **(A)** Representative images of colony formation and SA-β-gal staining of cells stably expressing plenti6.3 and plenti6.3-miR-139. **(B)** mRNA levels of miR-139-5p and senescence markers p15, p16, and p21 in HCC cells overexpressing miR-139. **(C)** Representative images of tumors in nude mice subcutaneously inoculated with BEL7404 cells stably expressing plenti6.3 and plenti6.3-miR-139 (seven mice in each group). (**D** and **E**) The tumor volume **(D)** and weight **(E)** in nude mice subcutaneously inoculated with BEL7404 cells stably expressing plenti6.3 and plenti6.3-miR-139. **(F)** Representative images of H&E, Ki67, TOP2A, and SA-β-gal staining of tumor tissues. **(G)** The cell rate of Ki67-positive cells in tumors. **(H)** The cell rate of TOP2A-positive cells in tumors. **(I)** The cell rate of SA-β-gal-positive cells in tumors. **(J)** mRNA levels of miR-139-5p, TOP2A, and senescence markers p15, p16, and p21 in tumors in nude mice subcutaneously inoculated with BEL7404 cells. *p < 0.05, **p < 0.01, ***p < 0.001

To investigate the function of miR-139-5p in HCC, we transfected HCC cells with miR-139-5p mimic and inhibitor. CCK8 and EdU assays showed that miR-139-5p mimics inhibited the viability and proliferation rate of HCC cells, whereas the miR-139-5p inhibitor promoted HCC cell proliferation (Fig. S6A and S6B). We then assessed the mRNA levels of cellular senescence markers and found that compared to the control group, p15 and p16 were upregulated in cells transfected with miR-139-5p mimics, whereas p15, p16, and p21 were downregulated in cells transfected with the miR-139-inhibitor (Fig. S6C). We constructed two HCC cell lines, BEL7404 and SMMC7721, with stable miR-139-5p overexpression (Fig. S6D). Overexpression of miR-139-5p inhibited colony formation and promoted senescence in HCC cells (Fig. 6A). The mRNA levels of p15, p16, and p21 in HCC cells were upregulated by miR-139-5p overexpression (Fig. 6B). Consistent with these in vitro results, the in vivo growth of xenografts derived from BEL7404 cells stably expressing miR-139-5p was significantly decreased (Fig. 6C and E). H&E, ki67, TOP2A, and SA-β-gal staining showed that ectopic miR-139-5p expression resulted in increased abnormal cell division, decreased cell proliferation activity, decreased TOP2A expression, and accelerated cellular senescence (Fig. 6F-I). Moreover, molecular markers of senescence were upregulated in miR-139-5p overexpressing tumors (Fig. 6J). These data suggest that miR-139-5p inhibits cell proliferation and induces cellular senescence in HCC cells.

### Clinical significance of the EZH2/miR-139-5p/TOP2A axis in HCC

**Fig. 7:**
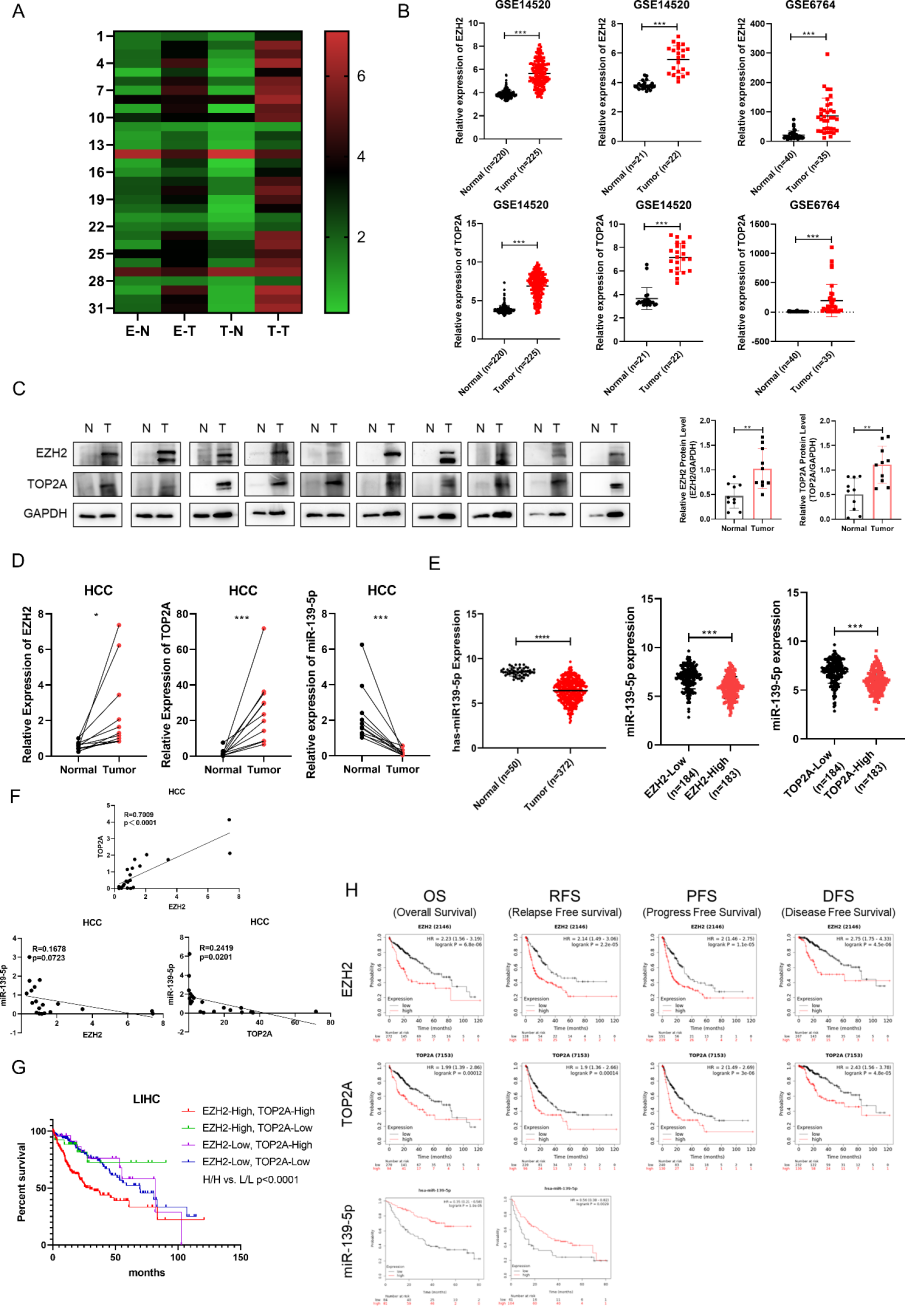
The expression profiles and correlation with prognosis of the EZH2/miR-139-5p/TOP2A axis in HCC. **(A)** The heatmap shows the expression profiles of EZH2 and TOP2A across tumor samples and paired normal tissues in 31 tumors in the TCGA database. E-N for EZH2 expression in normal tissues, E-T for EZH2 expression in tumor tissues, T-N for TOP2A expression in normal tissues, T-T for TOP2A expression in tumor tissues. **(B)** The expression of EZH2 and TOP2A in HCC cohorts (GSE14520 and GSE6764). **(C)** Protein levels of EZH2 and TOP2A in ten pairs of HCC tissues and para-carcinoma tissues. **(D)** mRNA levels of EZH2, TOP2A and miR-139-5p in ten pairs of HCC tissues and para-carcinoma tissues. **(E)** The expression of miR-139-5p in HCC in the TCGA database. **(F)** The expression correlation of EZH2, TOP2A and miR-139-5p in ten pairs of HCC tissues and para-carcinoma tissues. **(G)** Overall survival rate of HCC from the TCGA database analyzed according to the mRNA levels of EZH2 and TOP2A. HCC patients were divided into high and low groups according to the median mRNA expression level of EZH2 and TOP2A, respectively. HH for EZH2-High/TOP2A-High (n = 158); EZH2-High/TOP2A-Low (n = 27); EZH2-Low/TOP2A-High (n = 27); LL for EZH2-Low/TOP2A-Low (n = 158). **(H)** The correlation of EZH2, TOP2A, and miR-139-5p with overall survival (OS), relapse-free survival (RFS), progression-free survival (PFS), and disease-free survival (DFS) in patients with HCC. *p < 0.05, **p < 0.01, ***p < 0.001

We reanalyzed the expression data of EZH2 and TOP2A in tumor tissues and paired normal tissues from 31 tumors in the TCGA database and found that EZH2 and TOP2A were highly expressed in various solid tumors, including HCC (Fig. 7A). We then analyzed the expression levels of EZH2 and TOP2A in HCC and found that EZH2 and TOP2A were upregulated in HCC tissues compared to normal tissues in the GSE14520 and GSE6764 datasets (Fig. 7B). To further confirm these results, we collected ten pairs of HCC and adjacent normal tissues and found that EZH2 and TOP2A protein levels were higher in tumor tissues than in adjacent normal tissues (Fig. 7C). Consistently, compared with normal tissues, EZH2 and TOP2A mRNA levels increased in HCC tumor tissues and expression of miR-139-5p decreased in HCC tumor tissues (Fig. 7D). We analyzed the correlation of EZH2 and TOP2A with patients’ clinicopathological variables using HCC data from TCGA database and found that high EZH2 and TOP2A expression was significantly associated with tumor differentiation grade, tumor invasion, TNM stage, and the status of patients with HCC (Table 1). We further analyzed the data from TCGA database and confirmed the decreased expression of miR-139-5p in HCC. Compared with adjacent normal tissues, the miR-139-5p level is lower in HCC tumor tissues; compared with low-EZH2 HCC, miR-139-5p expression was lower in high-EZH2 HCC; compared with low-TOP2A HCC, miR-139-5p expression was lower in high-TOP2A HCC (Fig. 7E). Moreover, a positive correlation was found between EZH2 and TOP2A, whereas negative correlations were found between EZH2 and miR-139-5p and between TOP2A and miR-139-5p (Fig. 7F). To determine the prognostic value of the EZH2/miR-139-5p/TOP2A axis in HCC, we reanalyzed the prognostic data of HCC in the TCGA database and found that compared with patients with EZH2-low and TOP2A-low tumors, the prognosis of patients with EZH2-high and TOP2A-high tumors was worse (Fig. 7G). Furthermore, Kaplan–Meier curves showed that high EZH2, high TOP2A, and low miR-139-5p expression were correlated with adverse outcomes in patients with HCC (Fig. 7H). Taken together, our results reveal the expression profiles of the EZH2/miR-139-5p/TOP2A axis in HCC and a significant correlation between high EZH2, high TOP2A, and low miR-139-5p levels and poor prognosis in patients with HCC.

**Table 1:** Correlation between EZH2 and TOP2A expression and clinical characteristics in patients with HCC

Variables	Expression of EZH2		p	Expression of TOP2A		p
	High (n = 188)	Low (n = 187)		High (n = 188)	Low (n = 187)	
**Status**			0.002*			0.042*
Alive	106	135		111	130	
Dead	80	50		75	55	
**Age (years)**			0.198			0.032*
Age Mean (SD)	58.5 (12.9)	60.3 (14.0)		57.9 (13.1)	60.9 (13.8)	
Age Median [Min Max]	59 [18,85]	64 [16,90]		59 [18,85]	64 [16,90]	
**Gender**			0.196			0.083
Male	67	54		69	52	
Female	119	131		117	133	
**Grade of differentiation**			0.000***			0.000***
G1	17	38		17	38	
G2	78	99		73	104	
G3	78	44		84	38	
G4	10	2		9	3	
**Tumor invasion**			0.002*			0.002*
T1	72	109		75	106	
T2	59	35		55	39	
T3	48	32		48	32	
T4	7	6		8	5	
TX		1			1	
**Lymph node status**			0.345			0.264
N0	130	122		131	121	
N1	3	1		3	1	
NX	52	62		51	63	
**Distant metastasis**			0.476			0.586
M0	137	129		135	131	
M1	1	3		1	3	
MX	48	53		50	51	
**TNM stages**			0.024*			0.012*
I	69	102		15	38	
II	51	35		56	55	
III	53	32		82	68	
IV	1	4		18	20	

*p < 0.05, **p < 0.01, ***p < 0.001

## Discussion

As an epigenetic regulator, EZH2 promotes HCC progression by regulating stemness, chemosensitivity, and the tumor microenvironment [13–15]. However, the role of EZH2 and its mediated epigenetic modification in regulating cellular senescence in HCC remains unclear. In the present study, we found that EZH2 depletion induced senescence and inhibited proliferation of HCC cells both in vitro and in vivo. Increasing cellular senescence is the main feature of aging and a contributing factor in various age-related diseases, including cancer [30]. Multiple signaling pathways have been identified as hallmarks of senescence, including the DNA damage response, cyclin-dependent kinase inhibitors, cell cycle arrest, secretory phenotype, apoptosis resistance, metabolism, and endoplasmic reticulum stress [31]. CDK encoded in the CDKN2A (p16), CDKN2B (p15) and CDKN1A (p21) loci is the main driver of cell cycle arrest during senescence. Our results indicated that EZH2 downregulation resulted in an increase in senescent cells and upregulation of p15, p16, and p21. As the catalytic core of polycomb group complexes, EZH2 depletion induces senescence via two different mechanisms in normal cells during aging. Downregulation of EZH2 first initiates a DNA damage response and induces p21 expression, resulting in loss of H3K27me3 and induction of p16 expression in a DNA damage-independent manner [32]. However, the molecular mechanisms by which EZH2 regulates cellular senescence in tumors, especially HCC, remain largely unknown. In this study, we found that EZH2 inhibition induced cellular senescence and impaired cell proliferation in HCC via inhibition of H3K27me3-mediated repression of miR-139-5p and subsequent upregulation of TOP2A. A previous study indicated that EZH2 silencing-induced activation of p15 in cells without activation of p16 and p21 could induce cell senescence, indicating that cell type, tissue and species specificity are involved in the mechanisms of cellular senescence induction [33]. Our study uncovered the molecular mechanism of EZH2 and its epigenetic modification in regulating cellular senescence in HCC.

As a mitotic gene, TOP2A is essential for maintaining chromatin stability and regulating the cell cycle [34]. Aberrant expression of TOP2A induces cell cycle arrest and cellular senescence in HCC [25]. Our study showed that downregulation of TOP2A induced cellular senescence and inhibited the proliferation of HCC cells both in vitro and in vivo. Previous studies have identified dual upregulation of EZH2 and TOP2A as biomarkers for early identification of increased metastatic potential and recurrence among patients undergoing radiotherapy for prostate cancer [22, 35]. However, the regulatory mechanism of EZH2 and TOP2A remains unclear. In the present study, we found that both pharmacological inhibition and gene intervention of EZH2 inhibited TOP2A expression, indicating a positive regulatory relationship between EZH2 and TOP2A. We analyzed the ChIP-seq data of EZH2 and H3K27me3 from the ENCODE database and found no binding peak of EZH2 and H3K27me3 in the TOP2A promoter region. These results indicate that EZH2 and its epigenetic events indirectly promote TOP2A expression.

miRNAs are endogenous small, noncoding RNAs that negatively regulate protein-coding mRNA expression post-transcriptionally. A previous study reported that miRNAs are crucial mediators of EZH2 function in carcinogenesis [36]. Therefore, we screened for miRNAs that mediate the regulation of TOP2A by EZH2. We identified TOP2A as a novel, direct target of miR-139-5p. MiR-139-5p recognizes and combines with the 3′UTR of TOP2A and represses TOP2A expression. Consistent with a previous report on EZH2-mediated epigenetic silencing of miRNAs [36], we verified the EZH2-mediated epigenetic inactivation of miR-139-5p. EZH2-mediated H3K27me3 inhibits the expression of miR-139-5p, while knockdown of SUZ12 and EED, two PRC2 complex subunits, increased miR-139-5p expression. The results of the rescue assay showed that the loss of miR-139-5p compensated for the reduced expression of TOP2A induced by EZH2 inhibition. Collectively, these results indicate that miR-139-5p is an essential mediator of EZH2-mediated H3K27me3 and TOP2A expression.

MiR-139-5p is a well-characterized tumor-suppressing miRNA [37, 38]. In HCC, miR-139-5p inhibits aerobic glycolysis, cell proliferation, migration, and invasion by directly targeting the transcription factor ETS1 [39]. However, the role of miR-139-5p in cellular senescence has not been reported. In our study, in vitro gain- and loss-of-function experiments showed that miR-139-5p inhibited HCC cell proliferation by inducing cellular senescence and in vivo tumorigenesis assays indicated that miR-139-5p suppressed tumorigenicity. Moreover, miR-139-5p is downregulated in HCC tumor tissues, and low expression of miR-139-5p is correlated with adverse outcomes in patients with HCC. These results reveal the role of miR-139-5p in regulating cellular senescence in HCC, which enriches its function as a potent tumor suppressor and suggests that it might be a potential prognostic biomarker and therapeutic target for HCC.

EZH2 is overexpressed in a variety of tumor tissues, and high EZH2 expression is positively correlated with clinicopathological features and poor prognosis of cancers [40, 41]. TOP2A is upregulated in pancreatic cancer, and its upregulation is associated with tumor metastasis and shorter survival [42]. The present study revealed that EZH2 and TOP2A mRNAs and proteins were overexpressed in HCC tumor tissues, and their overexpression was correlated with differential tumor grade, tumor invasion, TNM stage, and shorter survival in patients with HCC. Moreover, TOP2A is more highly expressed in EZH2-high tumor tissues than in EZH2-low tumor tissues and normal tissues, and the coordinated expression of EZH2 and TOP2A is associated with a worse prognosis in HCC. This suggests that HCC with high EZH2 and TOP2A expression is more aggressive, thus revealing a therapeutic and prognostic role for the combination of EZH2 and TOP2A. To date, different types of EZH2 inhibitors have been developed and multiple clinical trials of drugs targeting EZH2 in different types of cancers are underway [7]. TOP2A inhibitors combined with the neddylation inhibitor MLN4924 show a synergistic effect in glioblastoma [43]. Given the coordinated expression of EZH2 and TOP2A in HCC, the combined use of EZH2 and TOP2A inhibitors might improve treatment efficacy and overcome the limitations of monotherapy.

Cellular senescence is a phenotype that steadily exposes tumor weaknesses once been induced and the physiology of senescent cells is unique in terms of metabolome, secretion, transcriptome, and epigenetics, so the vulnerability exposed is more selective. Induction of cancer cell senescence combined with a second drug that selectively eliminates senescent cancer cells, named “one-two punch” sequential treatment, may be a promising treatment strategy for cancer [44]. The “one-two punch” treatment model was proposed for HCC. The “first punch” is to use the specific mutations existing in tumor cells to induce them to a specific state - the state of cell senescence, so that tumor cells reveal “flaws”. The “second punch” accurately removes senescent cancer cells [45]. In the present study, we found that inhibition of EZH2 induced HCC cell senescence by disturbing H3K27me3/miR-139-5p/TOP2A signaling pathway. These results suggest that targeting EZH2 can directly inhibit the proliferation of liver cancer cells on the one hand, and induce senescence of HCC cells on the other hand. These induced senescent HCC cells can be selectively targeted by senolytic therapy. Moeover, evidence suggests that senescence induction therapies may have a synergistic effect with immunotherapies in cancer treatment [46]. EZH2 plays an important role in the immune microenvironment of HCC by regulating T-cell function, immune evasion, and NK-cell-mediated cytotoxicity [47]. Thus, targeting EZH2 may contribute to the synergistic anti-tumor efficacy of senescence induction therapies and immunotherapies.

In summary, we demonstrated the involvement of the EZH2/miR-139-5p/TOP2A axis in the regulation of cellular senescence and cell proliferation in HCC. EZH2, the catalytic core of the PRC2 complex, promotes the expression of TOP2A through H3K27me3-mediated epigenetic silencing of miR-139-5p. This finding reveals the mechanism of EZH2-mediated epigenetic modification in the regulation of senescence in HCC. The concurrent upregulation of TOP2A and EZH2 predicts a worse prognosis in patients with HCC, suggesting a therapeutic strategy for HCC that targets EZH2 to induce cellular senescence and then destroy senescent cells.

### Electronic supplementary material

Below is the link to the electronic supplementary material.

Supplementary Material 1

## Data Availability

The datasets used and analysed during the current study are available from the corresponding author on reasonable request.

## List of Abbreviations

ChIP: Chromatin immunoprecipitation
ChIP-seq: Chromatin immunoprecipitation sequencing
EZH2: Enhancer of zeste homolog 2
H&E: Hematoxylin and eosin
HCC: Hepatocellular carcinoma
IHC: Immunohistochemistry
IRF1: Interferon regulatory factor 1
NC: Negative control
SA-β-gal: Senescence-associated β-galactosidase
TCGA: The Cancer Genome Atlas
TOP2A: DNA topoisomerase II alpha
