# Supplementary material for: EZH2-H3K27me3-mediated silencing of mir-139-5p inhibits cellular senescence in hepatocellular carcinoma by activating TOP2A
Source: J Exp Clin Cancer Res. 2023 Nov 27;42:320. doi: 10.1186/s13046-023-02855-2 (PMC10680220; doi:10.1186/s13046-023-02855-2)
Supplement: Supplementary file 1 — Supplementary Material 1 [file 13046_2023_2855_MOESM1_ESM.pdf]

EZH2-H3K27me3-mediated Silencing of miR-139-5p Inhibits Cellular Senescence in Hepatocellular Carcinoma by Activating TOP2A

Ke Wang[1, 2, 3†], Xunliang Jiang[1, 3†], Yu Jiang[4†], Jun Liu[3], Yongtao Du[1, 3], Zecheng Zhang[1, 3], Yunlong Li[1], Xinhui Zhao[5], Jipeng Li[1, 6*], Rui Zhang[3, 7*]

Supplemental figures 1-6

Supplemental tables 1, 2

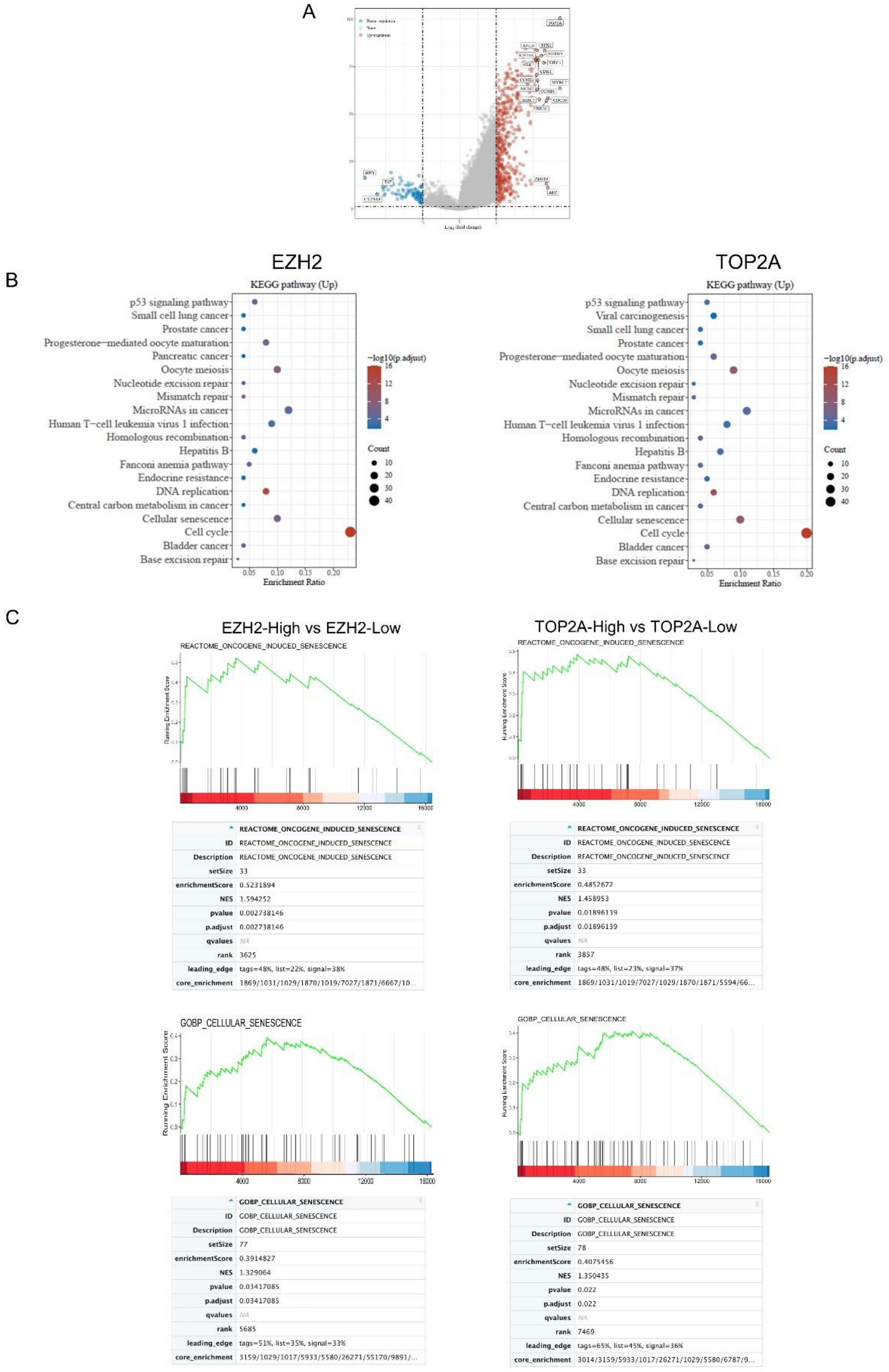

Figure S1. Correlation of EZH2, TOP2A and cellular senescence. (A) The Volcano Plot shows the differentially expressed genes of TOP2A in HCC by reanalyzing the

RNA-seq data in TCGA dataset using the limma package in the R software. (B) The KEGG enrichment analysis of upregulated differential genes of EZH2 and TOP2A in HCC. (C) The GSEA of RNA-seq data from TCGA of EZH2 high expression versus EZH2 low expression and TOP2A high expression versus TOP2A low expression using the Reactome oncogene induced senescence gene set annotated in the R-HSA-2559585 and GOBP replicative senescence gene set annotated in the GO: 0090399.

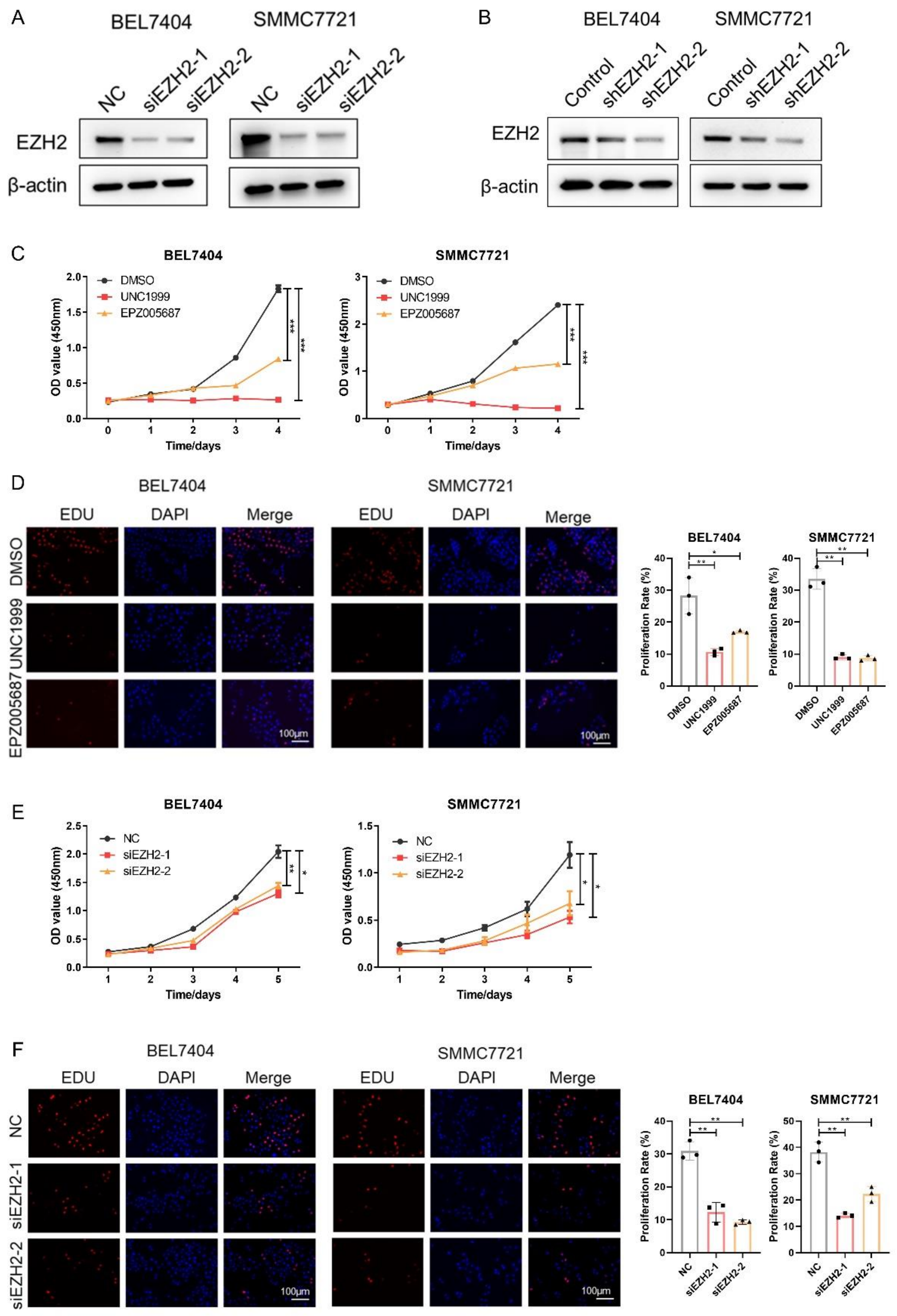

Figure S2. Inhibition of EZH2 suppresses proliferation of HCC cells. (A and B) Western blot analysis shows that EZH2 was knocked down by targeted siRNAs (A) and

shRNAs (B). (C and D) CCK8 shows that cell viability was decreased by EZH2 inhibition using EZH2 inhibitors UNC1999 and EPZ005687 (C) and targeted siRNA (D). (E and F) EdU assay shows that cell proliferation rate was reduced by EZH2 inhibition using EZH2 inhibitors UNC1999 and EPZ005687 (E) and targeted siRNA (F). *$p<0.05$, **$p<0.01$, ***$p<0.001$.

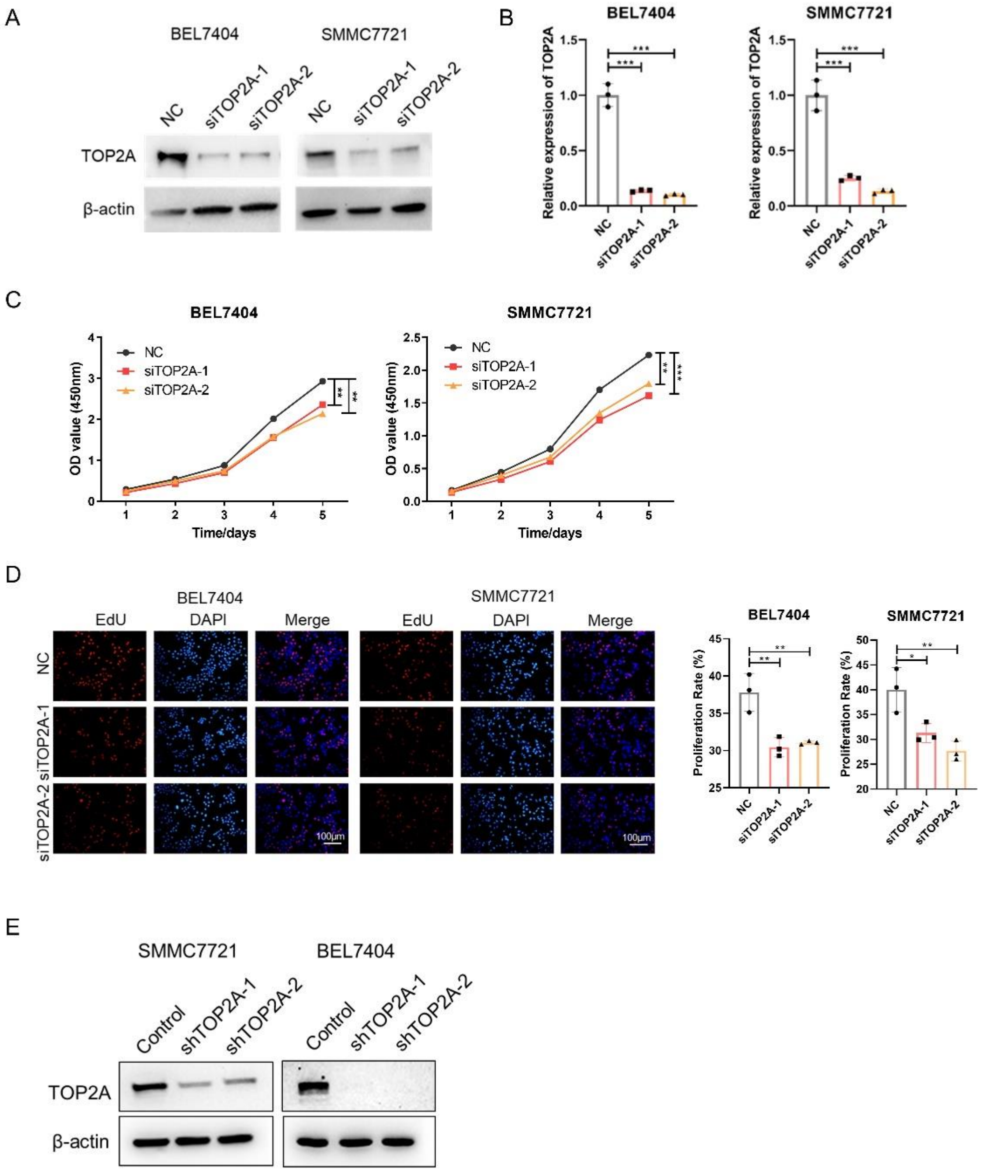

Figure S3. TOP2A Knockdown inhibits proliferation of HCC cells. (A and B) Western blot analysis (A) and RT-qPCR (B) of TOP2A in cells transfected with NC siRNA and TOP2A siRNAs. (C) Cell viability detected by CCK8 of cells transfected with NC siRNA and TOP2A siRNAs. (D) Cell proliferation ability tested by EdU aggay after cells transfected with NC siRNA and TOP2A siRNAs. (E) Western blot analysis of TOP2A in cells stable expressing plko.1-control and plko.1-shTOP2As. *p<0.05, **p<0.01, ***p<0.001.

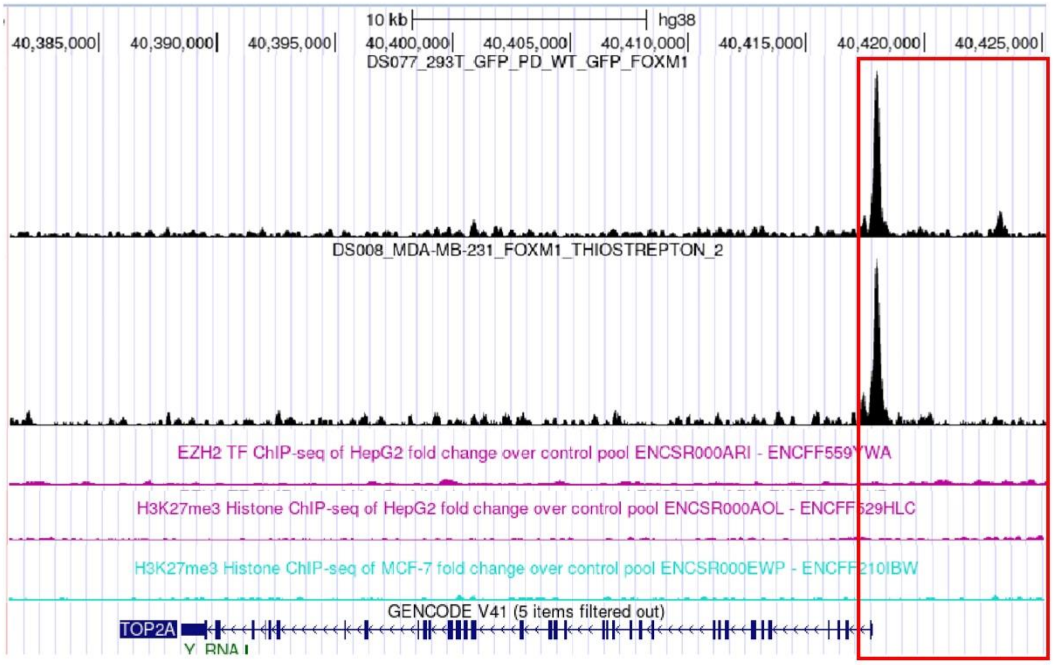

Figure S4. There is no binding peak of EZH2 and H3K27me3 on the TOP2A genomics locus. Analysis of the ChIP-seq data of EZH2 and H3K27me3 in HepG2 and MCF-7 cells and FOXM1 in HEK293T and MDA-MB-231 cells from the ENCODE database, respectively. The red box shows the binding peaks of FoxM1, EZH2, and H3K27me3 on the TOP2A genomic locus. FOXM1 was used as a positive control.

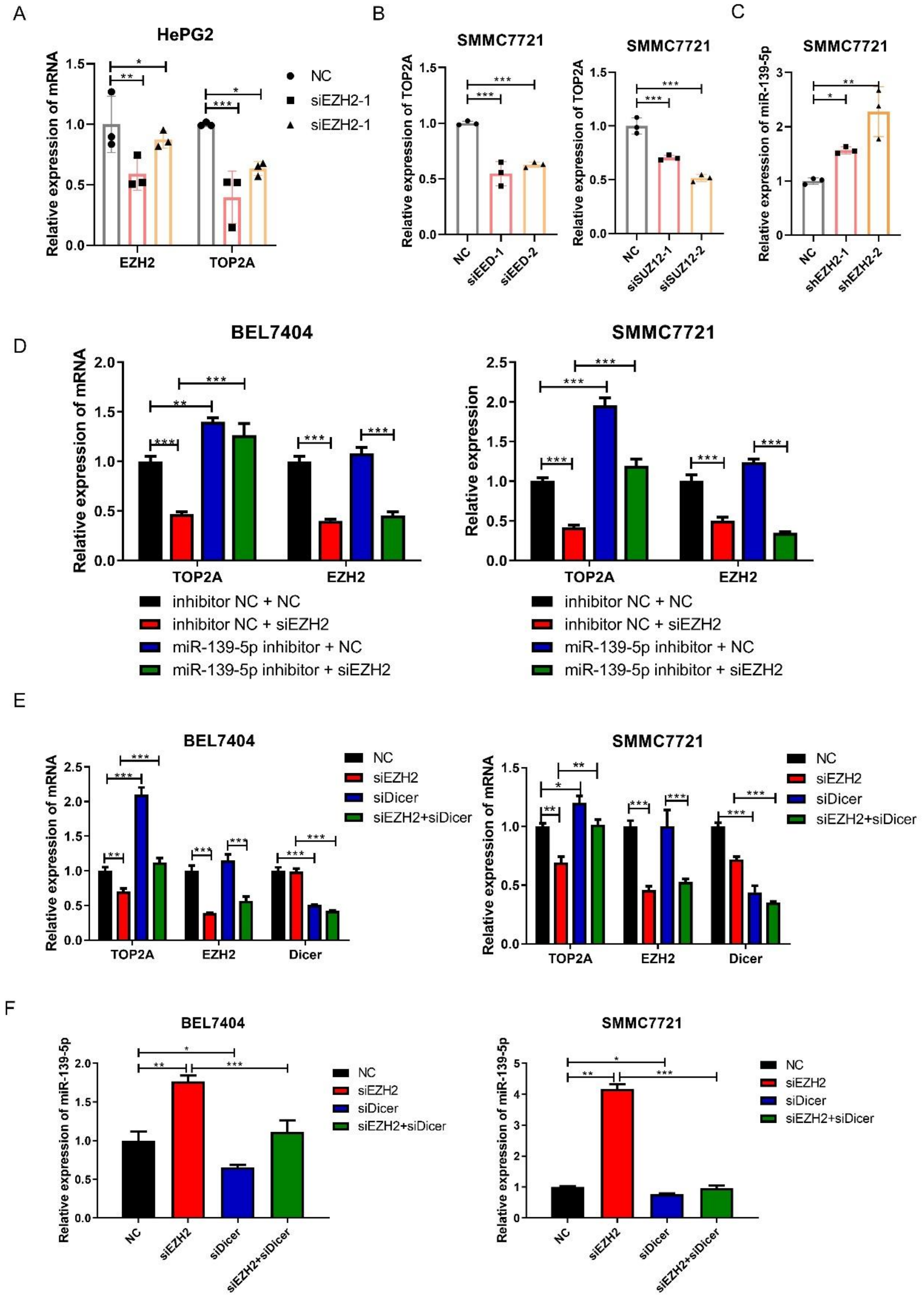

Figure S5. EZH2 acts as a positive regulator of TOP2A by promoting H3K27me3-mediated epigenetic silence of miR-139-5p. (A) mRNA levels of TOP2A and EZH2

in hepG2 cells transfected with EZH2 targeted siRNAs. (B) mRNA levels of TOP2A in SMMC7721 cells transfected with EED and SUZ12 targeted siRNAs. (C) mRNA levels of miR-139-5p in SMMC7721 cells stable expressing plko.1-control and plko.1-shEZH2s. (D) mRNA levels of TOP2A and EZH2 in BEL7404 and SMMC7721 cells transfected with inhibitor NC + siRNA NC, inhibitor NC + siEZH2, miR-139-5p inhibitor + siRNA NC, miR-139-5p inhibitor + siEZH2. (E and F) mRNA levels of TOP2A, EZH2, Dicer (E), and miR-139-5p (F) in BEL7404 and SMMC7721 cells transfected with siRNA NC, siEZH2, siDicer and siEZH2 + siDicer. *$p<0.05$, **$p<0.01$, ***$p<0.001$.

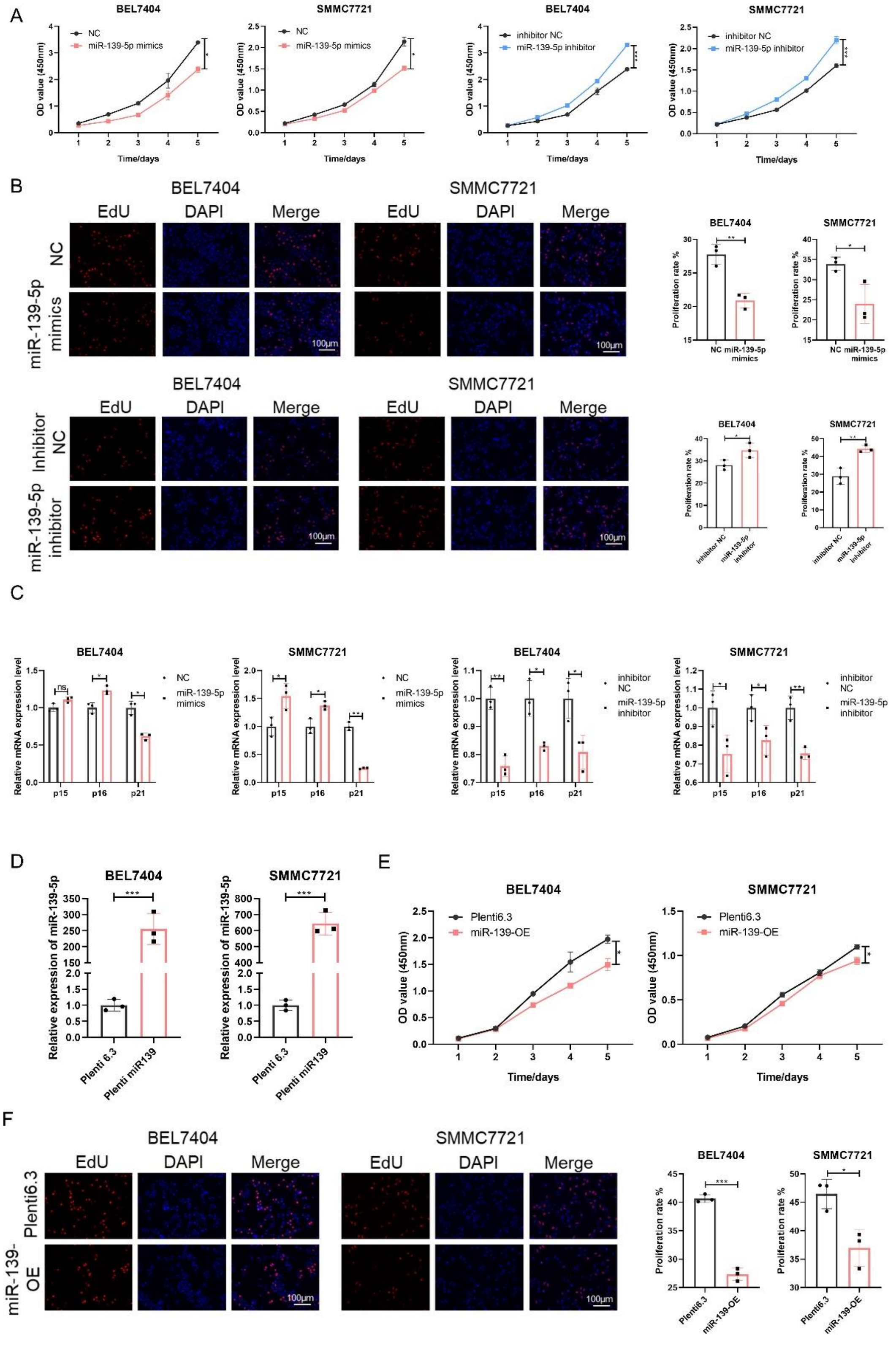

Figure S6. miR-139-5p impairs growth and induces senescence in HCC cells. (A) Cell viability of cells transfected with miR-139-5p mimics and inhibitor detected by

CCK8. (B) Cell proliferation rate of cells transfected with miR-139-5p mimics and inhibitor detected by EdU assay. (C) mRNA levels of senescence markers p15, p16, and p21 in cells transfected with miR-139-5p mimics and inhibitor. (D) mRNA levels of miR-139-5p in cells stable expressing plenti6.3 and plenti6.3-miR-139. (E) Cell viability of cells with miR-139 overexpression detected by CCK8. (F) Cell proliferation rate of cells with miR-139 overexpression detected by EdU. *$p<0.05$, **$p<0.01$, ***$p<0.001$.

Table. S1 siRNA sequences

siRNA	sense (5'-3')	antisense (5'-3')
siEZH2-1	GAAAGAUCUAGAGGAUAAUTT	AUUAUCCUCUAGAUCUUUCTT
siEZH2-2	GGAUGGUACUUUCAUUGAATT	UUCAAUGAAAGUACCAUCCTT
siTOP2A-1	GACUGUCUGUUGAAAGAAUTT	AUUCUUUCAACAGACAGUCTT
siTOP2A-2	CUCCUAACUUCUAGUAACUTT	AGUUACUAGAAGUUAGGAGTT
siDicer1	GGCUUACCUUCUCCAGGCUTT	AGCCUGGAGAAGGUAAGCCUU
siEED-1	AAGCACUAUGUUGGCCAUGGATT	UCCAUGGCCAACAUAGUGCUUTT
siEED-2	UGGUGCUGCUAUUCGACAATT	UUGUCGAAUAGCAGCACCATT
siSUZ12-1	GUCGCAACGGACCAGUUAATT	UUAACUGGUCCGUUGCGACTT
siSUZ12-2	GACUACAGAUCUACAAACATT	UGUUUGUAGAUCUGUAGUCTT
NC	UUC UCCGAACGUGUCACGUTT	ACGUGACACGUUCGGAGAATT
miR-139-5p-mimics	UCUACAGUGCACGUGUCUCCAGU	UGGAGACACGUGCACUGUAGAUU
inhibitor-NC	CAGUACUUUUGUGUAGUACAA	
miR-139-5p-inhibitor	ACUGGAGACACGUGCACUGUAGA	

Table. S2 qRT-PCR primers

qRT-PCR primers	
EZH2-F	AATCAGAGTACATGCGACTGAGA
EZH2-R	GCTGTATCCTTCGCTGTTTCC
TOP2A-F	ACCATTGCAGCCTGTAAATGA
TOP2A-R	GGGCGGAGCAAAATATGTTCC
p15-F	AAGCTGAGCCCAGGTCTCCTA
p15-R	CCACCGTTGGCCGTAAACT
p16-F	CCCAACGCACCGAATAGTTA
p16-R	ACCAGCGTGTCCAGGAAG
p21-F	CAGCAGAGGAAGACCATGTG
p21-R	GAGGCACAAGGGTACAAGACA
EED-F	GTGACGAGAACAGCAATCCAG
EED-R	TATCAGGGCGTTCAGTGTTTG
SUZ12-F	AGGCTGACCACGAGCTTTTC
SUZ12-R	GGTGCTATGAGATTCCGAGTTC
Dicer1-F	AAAATTGTCCATCATGTCCTCGC
Dicer1-R	CCACCAGGTCAGTTGCAGTT
miR-139-5p-F	TCTACAGTGCACGTGTCTCCAGT
U6-F	CTCGCTTCGGCAGCACA
